# N-Alkylamino Stilbene Compounds as Amyloid β Inhibitors for Alzheimer’s Disease Research

**DOI:** 10.3390/molecules30112471

**Published:** 2025-06-05

**Authors:** Citlali Gutiérrez, Liang Sun, Yiran Huang, Kai Gui, Karna Terpstra, Liviu M. Mirica

**Affiliations:** 1 Department of Chemistry, University of Illinois Urbana-Champaign, 600 S. Matthews Avenue, Urbana, IL 61801, USA; 2 The Neuroscience Program, Department of Bioengineering, Beckman Institute for Advanced Science and Technology, Carle Woese Institute for Genomic Biology, Carle Illinois College of Medicine, University of Illinois Urbana-Champaign, Urbana, IL 61801, USA

**Keywords:** Alzheimer’s disease, beta-amyloid, chemical tools, Aβ probes, protein aggregation

## Abstract

Alzheimer’s disease (AD) is an incurable neurodegenerative disorder that debilitates an overwhelming number of people in the aging population worldwide. The aggregated forms of the amyloid beta (Aβ) peptide play an important role in the onset of AD. Small molecules that can bind to Aβ are useful for in vitro assays, in vivo imaging, and in therapeutic research. Herein, a series of compounds that can target Aβ aggregates and inhibit their formation were developed. The interaction of several compounds with the Aβ peptide was found to modulate the formation of aggregates. These N-alkylamino stilbene compounds offer selectivity toward Aβ species against other in situ proteins and have potential for aiding the development of soluble Aβ aggregate selective AD probes.

## 1. Introduction

Alzheimer’s disease (AD) is a neurodegenerative disorder negatively impacting the cognitive functions of an ever-increasing number of people worldwide. First described in 1906 by Alois Alzheimer, the neuropathological markers can be described as either positive or negative lesions—the former containing amyloid plaques and neurofibrillary tangles and the latter describing cerebral atrophy [1]. Alzheimer’s disease remains incurable, and its unclear etiology remains a challenge for the development of effective diagnostics and treatments. Molecules that bind to AD biomarkers, such as the amyloid beta (Aβ) peptide that makes up amyloid plaques, are highly sought for use as a dye for in vitro assays, as a probe with in vivo imaging, and in therapeutic endeavors. Selectively targeting soluble Aβ oligomers is of particular interest due to their high neurotoxicity [2,3,4]. However, there remains limited understanding in the design of oligomer-selective probes. As such, probes that can inhibit insoluble amyloid aggregation may be used to investigate new compounds that selectively target soluble Aβ aggregates such as oligomers or protofibrils.

Small fluorescent molecules that bind to Aβ have long been inspired by chemical tools such as Congo Red (CR) and Thioflavin T (ThT) because of their great binding affinity to Aβ aggregates. Popular assay dyes, like ThT, contain a rotatable σ bond that, upon rotation, quenches their fluorescence. However, once bound to insoluble amyloid aggregates, i.e., fibrils or plaques, the dyes lock into a specific conformer, resulting in significant fluorescence enhancement. The structural moiety associated with the locked-conformation-dependent fluorescence enhancement phenomenon has been dubbed the molecular rotor motif or push-pull design. These molecular rotors perform twisted intramolecular charge transfer (TICT) from an electron-donating group along a highly conjugated π system to an electron-accepting group (D-π-A or D-A) [5]. This design concept has been applied to synthesize better binding fragments for extracellular amyloid plaques, one of the pathological hallmarks of the brains of people with Alzheimer’s disease [6]. However, more recent developments in the amyloid cascade hypothesis indicate that the deposition of amyloid plaques is not correlated with AD progression. More evidence has suggested that soluble Aβ oligomers, which precede amyloid plaque accumulation, are the first pathological change along the course progression of AD and are the most cytotoxic species [7,8,9]. Hence, chemical tools that can effectively inhibit the aggregation pathway of amyloids are essential for the improvement of more dyes selective for soluble Aβ.

Previously reported small molecule inhibitors for Aβ aggregation include compound classes such as curcuminoids (polyphenols) and flavonoids, whose structures have been shown to double as chelators for metal ion scavenging [10,11,12]. Stilbene-based inhibitors have also been reported, but they tend to suffer from any of the following: low Aβ selectivity, solubility issues, photosensitivity, and cellular toxicity [13,14]. The metal chelation ability of the previous classes can be incorporated onto the stilbene backbone by fusing a well-studied metal chelator such as a substituted 1,4,7-triazacyclononane (tacn). Previous works have investigated tacn-substituted stilbenes both by lengthening the π linker or modifying the linker electronic properties [15,16].

Herein, we report a novel series of compounds for which inhibition of the amyloid aggregation pathway was observed. Several compounds demonstrate potential within in vitro settings and for further development as chemical tools. As previously mentioned, a privileged stilbene scaffold is utilized as the conjugated π linker for these compounds [13,15,17,18,19,20,21,22,23,24,25,26]. The structural differences in these compounds were investigated to better inform the design of amyloid-targeting compounds in general. Accordingly, this series indicates important considerations for Aβ aggregate inhibitors to aid in the development of more selective AD probes.

## 2. Results

### 2.1. Design and Synthesis of Compounds

**Figure 1:**
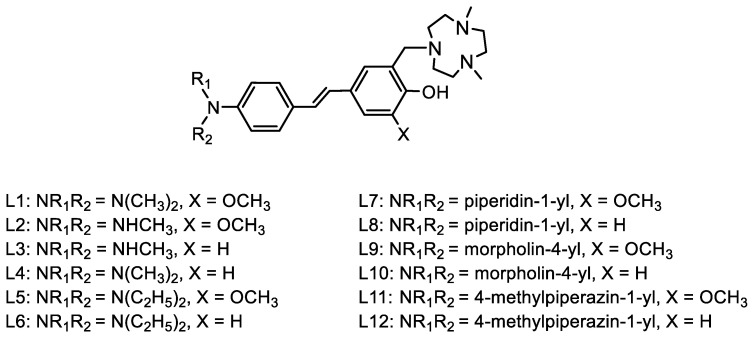
Structures of the derivatives labeled **L1**–**L12**.

The Aβ binding compounds contain an alkylamine moiety as the electron donor. These alkylamine-containing compounds have been previously used as electron-donating groups [27]. The stilbene, a privileged scaffold within amyloid binding, serves as the π framework that bridges the donor group to the acceptor group, a substituted phenol, as shown in Figure 1 [22]. While the phenol is not expected to be the best electron acceptor group, it has previously been shown to be a useful moiety for amyloid binding [16]. Rough approximations of electron-donating and electron-withdrawing properties for these compounds can be found in the Supplementary Materials (Table S5).

To better understand the structure–activity relationships of these stilbene-based dyes, the electron-donating (EDG) and electron-accepting (EAG) groups were derivatized. The Aβ-targeting stilbenes were synthesized using two different methods. The first method used followed the reported Kung procedure to successfully synthesize the mono- and di-methylamino stilbene derivatives [28]. The second method employed Heck reactions to generate the other stilbene products [29,30]. To synthesize the final compounds (**L1**, **L2**, **L3**, **L4**, **L5**, **L6**, **L7**, **L8**, **L9**, **L10**, **L11**, and **L12**), the Mannich reaction was performed among paraformaldehyde and an amine-containing fragment 2,4-dimethyl-1,4,7-triazacyclononane (tacn) with the stilbene derivatives.

Two classes of EDGs were synthesized [31]. The first type of donor group was monomethyl, dimethyl, or diethylamine. The second type of donor group was more rigid piperidine derivatives. The effect of heteroatom substitution on the para-position of the EDG piperidine was also examined. This series contains moderately effective EAGs that allow minor EDG electronic differences to be studied for better comparison. The two studied EAGs were tacn-substituted phenols in the presence or absence of an ortho-methoxy group.

### 2.2. Fluorescence Turn-On Effect of Compounds Interacting with Aβ Species

**Figure 2:**
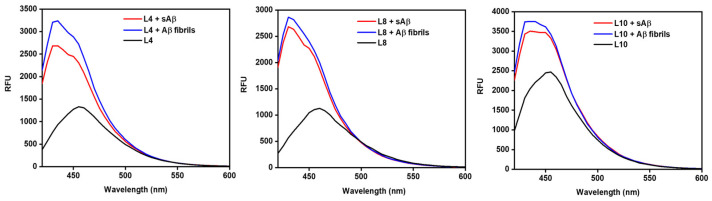
Fluorescence spectra of **L4** (**left**), **L8** (**middle**), and **L10** (**right**). **L4** is representative of the three compounds with the highest fluorescence intensities. **L8** has the highest fluorescence turn-on from baseline fluorescence. **L10** has the highest fluorescence intensity. [Compound] = 5 μM in PBS (pH = 7.4) with less than 10% DMSO for solubility. All fluorescence spectra found in Supplementary Materials Figure S1.

A fluorescence turn-on assay was performed to interrogate the effect of structural differences on the fluorescence enhancement with Aβ aggregates. These compounds showed a range of low-to-moderate background fluorescence intensity. Increases in fluorescence intensity with different aggregates would be ideal for tools used in amyloid-based assays. For both the methyl and dimethyl EDGs, **L1** and **L2**, the presence of an ortho-methoxy group on the EAG shows a slightly higher fluorescence enhancement with soluble amyloid beta (sAβ) over fibrils. **L2**, with a monomethylamine EDG, shows greater fluorescence turn-on than **L1**, which has a dimethyl amine EDG. Without the ortho-methoxy group on the EAG, there is greater fluorescence enhancement for Aβ fibrils over sAβ for **L3** and **L4**. The compounds containing a diethyl-amine EDG, **L5** and **L6**, show slightly greater turn-on with sAβ species, and the compound containing an ortho-methoxy on the EAG shows no discernible difference. The larger fluorescence enhancement observed for **L6** than **L5** indicates the dimethylamine EDG/methoxy EAG pair has a greater TICT. The piperidine-EDG-containing **L7** has only slightly higher turn-on with sAβ, which may be attributed to the ortho-methoxy group on the EAG. **L8** does not have that methoxy group and exhibits slightly greater turn-on for Aβ fibrils. In this case, it appears that the methoxy on the EAG diminishes the overall fluorescence of the compound since **L8** has greater baseline fluorescence and turn-on than **L7**. This is also the case for the morpholine-EDG-containing **L9** and **L10**. The lack of a methoxy group on the **L10** EAG, in conjunction with the oxygen heteroatom from the morpholine EDG, electronically favors turn-on with Aβ fibrils to a slight extent. However, for **L9**, the addition of a methoxy group onto the EAG shows equal turn-on to both sAβ and Aβ fibrils. Changing the EDG to a methyl piperazine, **L11**, increases baseline fluorescence and turn-on compared to **L9**. Interestingly, the addition of an o-methoxy group for **L9** does not follow the previous trend and instead diminishes overall fluorescence. **L11** and **L12**, both containing an N-methyl piperazine EDG, show similar turn-on with sAβ and fibrils, and the o-methoxy EAG of **L11** increases overall baseline fluorescence compared to **L12**. **L4**, **L8**, and **L10** have the greatest overall fluorescence enhancements, with **L8** showing the greatest fluorescence enhancement of these compounds (Figure 2). The spectra for the remaining compounds can be found in the Supplementary Information (Figure S1). None of the three compounds with the highest fluorescence contained an o-methoxy group on the EAG.

The fluorescence properties of these compounds suggest that, at least for these stilbenes, the substitution of o-tacn onto the phenol EAG does not appreciably increase interactions with sAβ to cause greater turn-on, unlike previously reported results [16]. Notably, the fluorescence enhancement is modulated, either increasing or decreasing depending on the compound, by the addition of a methoxy substituent on the EAG. Finally, the fluorescence turn-on appears to depend on the EDG–EAG pair instead of the individual strength of electron donation and acceptance of each group, respectively.

### 2.3. Aβ Aggregation Inhibition Assays

**Figure 3:**
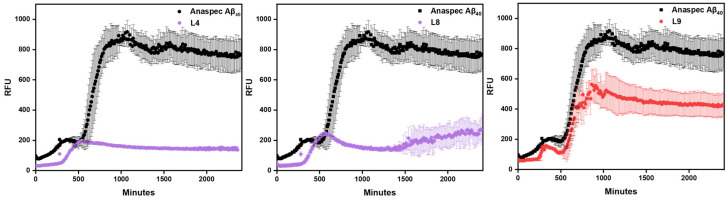
Amyloid aggregation inhibition of **L4** (**left**), **L8** (**center**), and **L9** (**right**). **L4** shows the greatest inhibition, whereas **L9** shows the least inhibition. All spectra found in Supplementary Materials Figure S2.

To investigate the effect of these compounds on amyloid aggregation, inhibition of aggregation assays were performed using the Aβ_40_ peptide. Aβ_40_ has been implicated in inducing oxidative stress and is physiologically the most common isoform of the amyloid peptide and is therefore a widely used isoform in amyloid assays [32,33]. Aβ_40_ is also known to have slower aggregation kinetics than Aβ_42,_ which is helpful for studying the inhibition of the early aggregation pathway [34]. These experiments track the time duration of fibril growth, which is monitored by ThT fluorescence turn-on, represented by relative fluorescence unit (RFU) intensity. A lower RFU signifies a lack of soluble amyloid species as ThT binds to insoluble forms. Thus, a lower RFU can be interpreted as inhibiting the fibrillization of amyloids. **L4** and **L8** showed the best inhibition of amyloid aggregates as shown in Figure 3. The rest of the compounds show appreciable inhibition of Aβ aggregation as found in the Supplementary Materials (Figure S2), except for **L9**, which contains the morpholine EDG and a methoxy group on the EAG (Figure 3). The addition of the more polar oxygen heteroatom may instead favor hydrogen bonding interactions within the aqueous media, thereby solvating the compound and reducing early interactions with soluble aggregates. Lastly, although **L2** and **L3** show similar amyloid inhibition, the **L3** compound lacking the o-methoxy group begins inhibition at an earlier time.

Likewise, based on the inhibition assays, the methoxy EAG once again plays an important role in tuning amyloid inhibition for these compounds. For instance, the piperidine EDG derivatives with the methoxy group on the EAG show lesser or later inhibition of aggregation. Comparing the different EDGs of mono- and disubstituted amine scaffolds containing the same EAGs suggests a general trend of greater Aβ fibril inhibition for the compounds containing an EAG without the o-methoxy group.

### 2.4. Cytotoxicity of Compounds in N2a Cells

**Figure 4:**
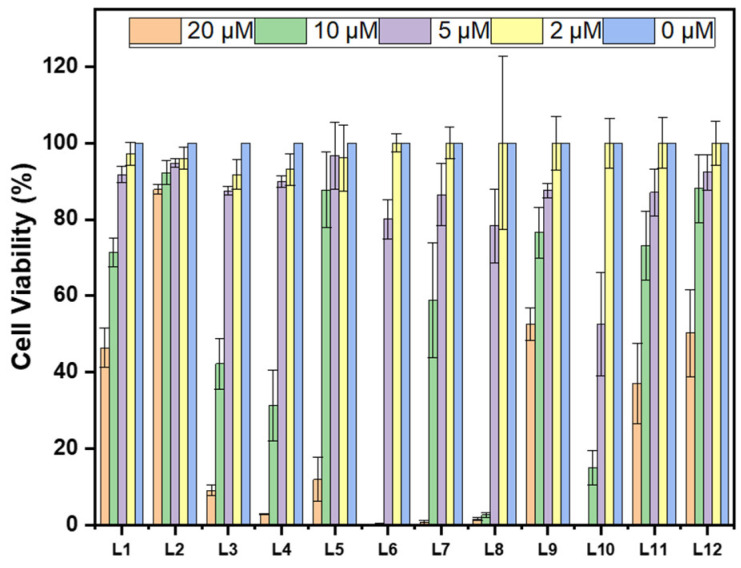
Toxicity of compounds **L1**–**L12** (clustered on the x-axis from left to right) at different concentrations (0, 2, 5, 10, and 20 μM) in N2a (mouse neuroblastoma) cells. Percent cell viability is shown on the y-axis. Intensities are normalized to DMSO-only control.

For in cellular assays, useful Aβ inhibitors would require low toxicity. The Alamar blue assay was used for the evaluation of the cytotoxicity of these stilbenes. **L2** has the highest cell viability at the highest tested concentration, 88%, while the rest show lower cell viability, as shown in Figure 4. The monomethyl and methoxy groups on **L2** may be attributed to reducing cytotoxicity in comparison to **L1** and **L3**. Of the cyclic amine EDG derivatives, **L9** and **L12** have the best cell viability. The oxygen heteroatom on **L9** does not play an important role in alleviating cell toxicity, but the addition of a methoxy group on this EDG–EAG system reduces toxicity compared to its **L10** analog. The extra methylated amine group from the piperazine EDG on **L11** and **L12** switches the cytotoxic effect of the methoxy group on the EAG, such that **L12** is less cytotoxic. The effect of the additional heteroatoms on toxicity is likely due to off target binding that induces cell death, although the mechanism is currently unclear. Interestingly, with the presence of the methoxy group, the cell viability increased compared to the counterpart compounds without that moiety, except for **L11** versus **L12**.

### 2.5. Effect of Inhibitors on Aβ_42_ Neurotoxicity in N2a Cells

**Figure 5:**
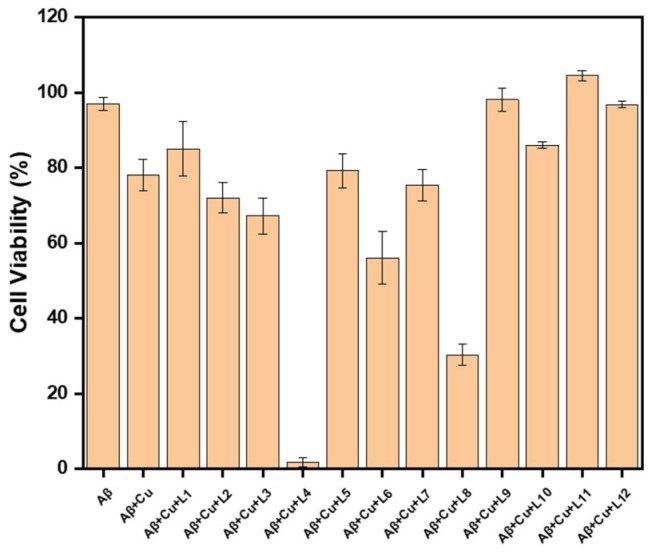
Effects of Aβ-inhibitors on Cu-induced Aβ_42_ cytotoxicity. Conditions: [Aβ_42_] = [CuCl_2_] = 20 μM, [**L1** to **L12**] = 5 μM. The reported cell viability % are relative to the DMSO control without Aβ_42_ or CuCl_2_.

These compounds can chelate metals via their substituted tacn moiety on the phenol EAGs, meaning that these inhibitors may also have utility in metal-involved assays. Copper is known to have deleterious effects within the brain environment due to its propensity to induce oxidative stress [35]. Therefore, we tested their ability to inhibit the Cu-mediated Aβ_42_ cytotoxicity on N2a cells. This procedure has been adapted from our previous publications after finding that in vitro conditions for Aβ toxicity are best achieved with the addition of copper [15,35,36,37]. As shown in Figure 5, **L9** and **L11** inhibit both Cu-stabilized Aβ_42_- and Aβ_42_-induced toxicity. The worst performing compounds were **L4** and **L8**, for which toxicity was exacerbated. Lastly, contrasting the toxicity of the EAG counterparts, it appears that the o-methoxy group assists in rescuing the cells from toxicity. Similarly, the methoxy-containing derivatives generally rescued cells from copper-induced toxicity. It is possible that the o-methoxy may rescue the Cu-stabilized Aβ_42_ toxicity in two ways, either via an o-vanillin-like antioxidant mode of action, or via improved Cu chelation. O-vanillin has previously shown antioxidant properties, which these compounds may emulate [38,39,40]. Cu chelation might be further improved with the methoxy arm, thereby increasing its copper binding affinity and reducing any Cu leaching [37,41].

In general, all compounds are well tolerated up to 5 μM. Specifically, **L1**, **L5**, **L9**, **L10**, **L11**, and **L12** rescue cells from Cu-Aβ_42_ cytotoxicity, likely by modulating the formation of off-pathway aggregates between Aβ and metal ions. Even so, both cell viability and Cu-Aβ-induced toxicity studies have shown that dimethyl and diethyl EDGs, **L1**–**L6**, have some level of toxicity associated with them, which is not found for the monomethyl, morpholine, and piperazine derivatives **L7**–**L12**, except for **L8**. The mechanism of the cytotoxicity of **L4** and **L8** with Cu and Aβ is not easily understood based on these studies. While the toxicity profiles of **L11**, **L10**, and **L5** are acceptable for in vitro studies, the Aβ_42_ inhibition profile is not the best from this set of ligands.

### 2.6. Fluorescence Imaging of Amyloid Plaques in 5xFAD Mice Brain Sections

To further investigate the affinity of the developed compounds toward various Aβ aggregates in situ, the intrinsic fluorescence properties of the compounds were employed for their ability to stain insoluble amyloid plaques. For these studies, brain sections from 11-month-old 5xFAD transgenic mice, which rapidly develop severe amyloid pathology, were co-stained with stilbenes and HJ3.4 antibody. HJ3.4 is an anti-amyloid antibody that binds to the N-terminal region of the Aβ peptide, thereby targeting both soluble and insoluble amyloid species [42,43,44].

**Figure 6:**
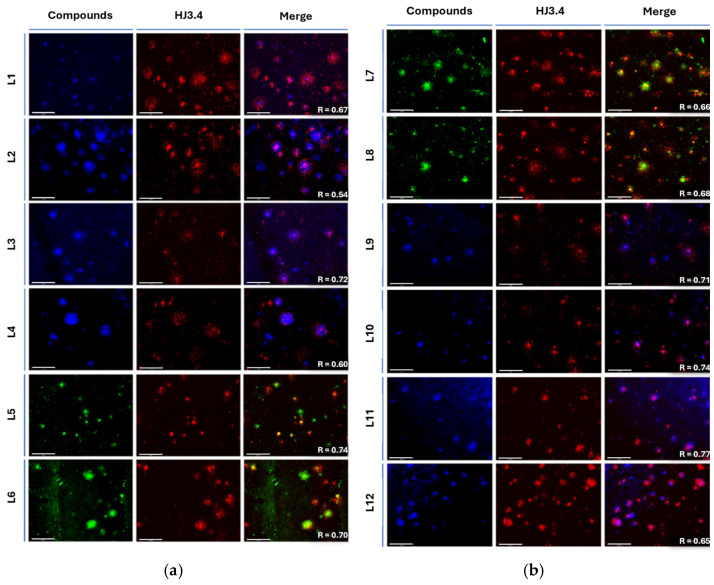
Fluorescence microscopy images of 5xFAD mice brain sections co-incubated with compound (25 μM, left image), AF594-HJ3.4 (1 μg/mL) (middle image), and merged images (right image): (**a**) compounds **L1** (R = 0.67), **L2** (R = 0.54), **L3** (R = 0.72), **L4** (R = 0.60), **L5** (R = 0.74), **L6** (R = 0.70) from top to bottom; (**b**) Compounds **L7** (R = 0.66), **L8** (R = 0.68), **L9** (R = 0.71), **L10** (R = 0.74), **L11** (R = 0.77), and **L12** (R = 0.65) from top to bottom. Scale bars on the lower left of each image measure 100 μm. Pearson’s coefficient (R) calculated using Fiji (ImageJ 1.52p).

Interestingly, for **L3** and **L4** a large amount of well-defined fluorescence staining was observed (Figure 6a, left image), and the plaques were also stained by AF594-HJ3.4 (middle panel), and the two sets of fluorescence images were well colocalized as analyzed by Pearson’s coefficient, confirming that the compounds efficiently accumulate into and label amyloid plaques specifically. However, with the introduction of the methoxy EAGs on the mono-EDG stilbene derivatives, the binding selectivity of the compounds marginally decreases, and non-specific binding increases, affecting the imaging of Aβ as Pearson’s coefficient decreases from R = 0.72 (**L3**) to R = 0.54 (**L2**). The dimethyl (R = 0.67 (**L1**) compared to R = 0.60 (**L4**) without OMe) and diethylamino (R = 0.74 (**L5**) compared to R = 0.70 (**L6**) without OMe) scaffold show better overall binding to amyloid and even greater Aβ selectivity with the methoxy EAG, whereas the piperidine EDG (R = 0.68 (**L8**) decreases to R = 0.66 (**L7**) with OMe) shows comparable results and similar trends to the monomethylamine structures with the addition of the methoxy group on the EAG. The morpholine EDG shows overall better selectivity to Aβ, but slightly better colocalization is shown without the methoxy EAG (R = 0.74 (**L10**) decreases to R = 0.71 (**L9**) with the OMe). The methyl piperazine EDG has good selectivity for Aβ species with the methoxy EAG but has slightly decreased selectivity without the methoxy group (R = 0.65 (**L12**) increases to R = 0.77 (**L11**) with the OMe). Positively, the in situ brain section staining shows good colocalization of all compounds, with slight differences in selectivity for Aβ species between those compounds modulated by the addition or lack of the methoxy on the EAG.

### 2.7. Docking Studies of Compounds on Aβ

**Figure 7:**
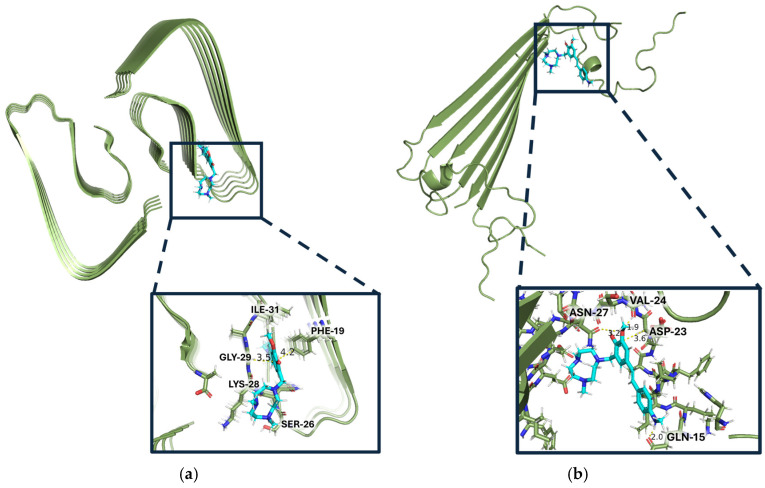
Binding modes of the compounds (shown in cyan) with the best docking scores to each type of model (shown in green), **L7** for insoluble amyloid and **L2** for soluble amyloid: (**a**) **L7** docked into binding pocket of fibril model PDB:5OQV; (**b**) **L2** binding pocket in PDB: 6RHY.

Finally, to gain understanding for how these Aβ inhibitors may interact with different Aβ_42_ aggregates, a series of docking studies were performed with Schrödinger Glide. Using the Aβ_42_ fibril structure (PDB ID: 5OQV) and the Aβ_42_ oligomer model (PDB ID: 6RHY), the most appropriate PDB model for soluble species to date, the compounds’ binding scores and binding pockets were investigated [45,46,47,48]. These PDBs were chosen because they most resemble a native conformation state for both fibrils and oligomers, respectively. Analysis for these compounds includes the docking score, an empirical score that approximates ligand binding free energy and considers Epik penalties [49,50,51]. The docking scores for each compound with both the Aβ_42_ fibril structure (5OQV) and the Aβ_42_ oligomer model (6HRY) can be found in Tables S2 and S3 in the Supplementary Materials. The best ligand for fibril docking (**L7** with docking score of −7.749 on 5OQV) respective to its binding pocket has a better docking score than the best ligand for soluble amyloid docking (**L2** with docking score of −5.055 on 6RHY) respective to its binding pocket, suggesting that these compounds are generally better binders of amyloid fibrils over any other aggregates. Interestingly, the Aβ fibril-docked compounds all have the same binding mode within the binding pocket, interacting with the FKGA amino acid sequence shown in Section S9 of the Supplementary Materials. The compound with the best docking score for 5OQV is **L7** (Figure 7a). **L7**, along with the next best docking compound for fibrils, **L11**, both have an ortho-methoxy on the EAG. These two compounds also have a third ring, piperidine and methyl piperazine, respectfully, that elongates the ligands. Based on the docking studies, increased length of the compound and a more planar EDG favor binding to the longer fibrillar structure, 5OQV.

Overall, these results confirm that the fluorescence assays are indicative of the electronic properties of the individual EDG and EAG displaying TICT behavior, since the binding pockets are different depending on the Aβ model. Since the docking grid encompasses the whole protein, the most energetically favorable location for the ligand to be docked was either alongside the beta sheet (6RHY) or fold (5OQV), or onto the protein periphery. The most favorable docking location for the compounds on 6RHY was not alongside the beta sheet, analogous to the beta fold of 5OQV, but rather on the periphery of the protein. The docking scores suggest that the length and planarity of the π system are important factors for binding to amyloid species. Despite these compounds being non-selective against soluble or insoluble aggregates, they provide insight for potential rules on aggregate selectivity and may prove valuable for experimental controls in search of compounds selective for soluble Aβ aggregates.

## 3. Discussion

### 3.1. Application of Amyloid Inhibitors

All these compounds are inhibitors of amyloid aggregation and can be used to modulate the formation of aggregates in vitro. In general, all compounds are well tolerated up to 5 μM, which may prove to be a limitation for most of them for in cellular experiments. While **L1**, **L5**, **L9**, **L10**, **L11**, and **L12** all rescue the cells from Cu-Aβ_42_ cytotoxicity, it is important to note that the role of metals such as copper will affect the suitability of these compounds for such related assays. The toxicity profiles of **L11**, **L10**, and **L5** show their utility for in cellular studies, but these three compounds are some of the lower performing amyloid inhibitors as indicated by the Aβ_42_ inhibition profile. In situ brain section staining shows good overlap between the compounds and the antibody control, signifying good selectivity for amyloid species. The log D values for these compounds, as shown in Table S4, indicate appreciable lipophilicity for blood–brain barrier crossing. There were no trends of log D associated with any one structural moiety. Unfortunately, despite the promising log D values, the toxicity profiles for most of these compounds are not ideal for in vivo applications. Based on the docking studies, it was determined that the increased length of the compound and a slightly more planar EDG favor binding to the longer fibrillar structure, 5OQV, and that, upon comparison with the docking scores for 6RHY, these compounds are generally more selective for the amyloid fibrils than any other aggregates. Overall, these results are informative for the design of amyloid-targeting inhibitors that can be advanced as chemical tools for the development of more selective soluble amyloid beta probes.

### 3.2. Interpretation of Docking Results

When docked onto the Aβ oligomer model, the binding pocket was different based on the compounds (Table S2). The compound with the best docking score onto oligomers (6RHY), **L2**, has a binding pocket consisting of the QVVNS amino acids (Figure 7b). According to molecular modeling, all compounds containing an o-methoxy group on the EAG exhibit a greater docking score onto the fibrils than their non-o-methoxy derivative counterparts, except for **L9** and **L10**. One explanation for this exception may be that these morpholine EDG-containing compounds have such unfavorable binding in one region due to the oxygen heteroatom that even the addition of a methoxy group on the EAG, while not overcoming these negative effects, may mitigate it, such that the fluorescence enhancement is not significantly different from the others (Figure S1). There was no such trend of the methoxy arm when docked upon oligomers, 6RHY, suggesting that the methoxy arm does not particularly stabilize interactions with soluble amyloid species as it does with fibrils.

The opportunity for π stacking interactions between the phenylalanine from Aβ_42_ and the styryl backbone of these compounds can explain why the docking scores for these compounds are more favorable with Aβ fibrils than soluble aggregates. In lieu of π stacking interactions, the Aβ oligomer-docked compounds primarily relied on hydrogen bonding interactions of the tacn moiety with the peptide backbone. Notably the tacn moiety does not permit the planar stilbene region of the compounds to bind deeply within the grooves of either Aβ structure as expected for TICT compounds such as ThT. The shorter beta sheet of the oligomer of 6RHY prevents π interactions, unlike the longer beta sheets of 5OQV fibrils, which better accommodates the stilbenes. All the while, the hydrophilic tacn moiety remains on the outskirts, weakly binding to the protein. Although the binding pocket was different from Aβ fibrils to oligomers, the compounds still had very similar turn-on for both Aβ oligomers and fibrils in the fluorescence studies. **L9**, despite being the worst amyloid inhibitor from the inhibition assay, is not predicted to be the worst of these compounds for either aggregate docking, suggesting it may interact with a form of soluble amyloid aggregate not studied herein, such as protofibrils [52,53,54].

## 4. Materials and Methods

### 4.1. General Experimental Details

All reagents were purchased from commercial sources and used as received unless stated otherwise. All solutions and buffers were prepared using metal-free Millipore water that was treated with Chelex resin overnight and filtered through a 0.22 μm nylon filter. ^1^H NMR (300.121 MHz) spectra were recorded on a Varian Mercury-300 spectrometer (Palo Alto, CA, USA). Chemical shifts were reported in ppm downfield from tetramethylsilane. UV−visible spectra were recorded on a Varian Cary 50 Bio spectrophotometer and were reported as λ_max_, nm (ε, M^−1^ cm^−1^). 5xFAD transgenic mice (Tg6799 line) overexpressing mutant human APP (695) with the Swedish (K670N, M671L), Florida (I716V), and London (V717I) mutations were purchased from Jackson Laboratories (Bar Harbor, ME, USA). All normal and C-18 reversed-phase column chromatography was performed on a Teledyne Isco Combiflash Rf+ (Thousand Oaks, CA, USA).

### 4.2. Synthetic Details

**Scheme 1:**
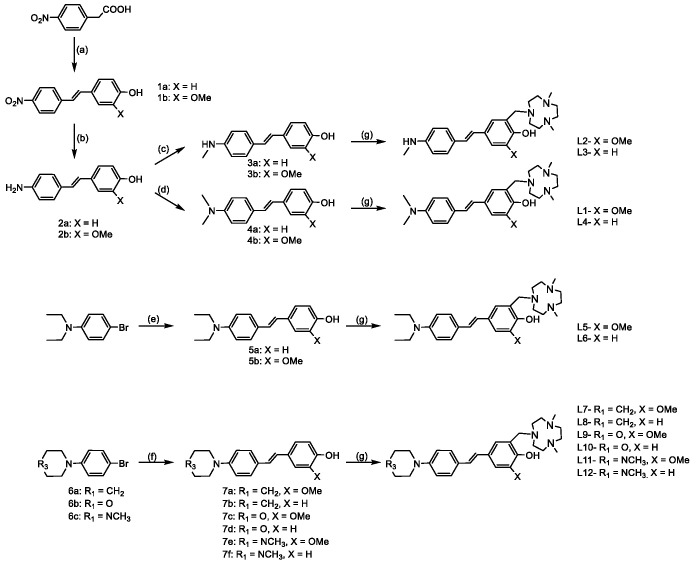
Synthesis of the compounds. (a) 4-hydroxybenzaldehyde or 4-hydroxy-3-methoxybenzaldehyde, piperidine, toluene, reflux, 3 h; (b) SnCl_2_, conc. HCl, EtOH, reflux, 3 h; (c) (CH_2_O)_n_, NaBH_4_, NaOMe, CH_3_OH (d) (CH_2_O)_n_, NaBH_3_CN, CH_3_COOH, room temperature, overnight; (e) 4-vinylphenol or 2-methoxy-4-vinylphenol, Pd(OAc)_2_, triethanolamine, 100 °C, 24 h; (f) 4-vinylphenol or 2-methoxy-4-vinylphenol, Pd(OAc)_2_, triethanolamine, 100 °C, 24 h; (g) Me_2_HTACN, (CH_2_O)_n_, MeCN, reflux, 24 h.

The full synthetic route is shown in Scheme 1, and precursors are labeled as indicated on the schematic.

#### 4.2.1. Synthesis of Precursors: **1a**, **1b**, **2a**, **2b**, **3a**, **3b**, **4a**, and **4b**

*Compound **1a** ((E)-4-(4-nitrostyryl)phenol) or **1b** ((E)-2-methoxy-4-(4-nitrostyryl)phenol).* A mixture of 2-(4-nitrophenyl) acetic acid (for **1a**: 500 mg, 2.8 mmol)(for **1b**: 2 g, 11 mmol), (for **1a**) 4-hydroxybenzaldehyde (337 mg, 2.8 mmol) or (for **1b**) 2-methoxy-4-vinylphenol (1.68 g, 11 mmol), and piperidine (for **1a**: 271 μL)(for **1b**: 1.1 mL) was added in toluene (**1a**: 50 mL, **1b**: 100 mL). The resultant mixture was heated to reflux for 2 h. The solvent was removed, and the resulting residue was purified by silica gel column chromatography using EtOAc/Hexane (1: 3) to yield a yellow solid.

**1a:** (418 mg, 63%); ^1^H NMR (DMSO-d6): *δ* (ppm): 9.77 (s, 1H), 8.18 (d, 2H, *J* = 8.4 Hz), 7.77 (d, 2H, *J* = 8.5 Hz), 7.49 (d, 2H, *J* = 8.2 Hz), 7.42 (d, 1H, *J* = 16.6 Hz), 7.16 (d, *J* = 16.4 Hz, 1H), 6.79 (d, *J* = 8.1 Hz, 2H).

**1b:** (1.8 g, 60%); ^1^HNMR (Acetone-d6): *δ* (ppm) 8.21 (d, 2H, *J* = 9.0 Hz), 7.79 (d, 2H, *J* = 8.8 Hz), 7.45 (d, 1H, *J* = 16.4 Hz), 7.34 (d, 1H, *J* = 2.0 Hz), 7.25 (d, 1H, *J* = 16.4 Hz), 7.14 (dd, 1H, *J* = 8.1, 2.0 Hz), 3.92 (s, 2H).

*Compound **2a** ((E)-4-(4-aminostyryl)phenol) or **2b** ((E)-4-(4-aminostyryl)-2-methoxyphenol)* [55]. Stannous chloride (9.3 mol) was added to a solution of compound **1a** or **1b** (1.9 mmol) in ethanol (100 mL), followed by the addition of concentrated hydrochloric acid (0.75 mL). The solution was brought to reflux for 3 h and cooled to room temperature, with stirring overnight. Saturated sodium bicarbonate was added to adjust the pH to 8–9. After standard workup with ethyl acetate, crude product **2a** or **2b** was obtained and was used in the following step without further purifications.

**2a:** (394 mg, ~100%); ^1^H NMR (DMSO-d6): *δ* (ppm): 9.46 (s, 1H), 7.28 (d, 2H, *J* = 8.7 Hz), 7.18 (d, 2H, *J* = 8.5 Hz), 6.78 (d, 2H, *J* = 2.6 Hz), 6.71 (d, 2H, *J* = 8.6 Hz), 6.52 (d, 2H, *J* = 8.5 Hz), 5.20 (s, 2H).

**2b:** (490 mg, ~100%); ^1^H NMR (Acetone-d6): *δ* (ppm): 7.59 (s, 1H), 7.27 (d, 2H, *J* = 8.5 Hz), 7.16 (d, 1H, *J* = 1.9 Hz), 7.00–6.89 (m, 2H), 6.86 (d, 1H, *J* = 16.3 Hz), 6.79 (d, 1H, *J* = 8.1 Hz), 6.66 (d, 2H, *J* = 8.6 Hz), 4.74 (s, 1H), 3.88 (s, 3H).

*Compound **3a** ((E)-4-(4-(methylamino)styryl)phenol) or **3b** ((E)-2-methoxy-4-(4-(methylamino)styryl)phenol).* To a mixture of **2a** or **2b** (0.5 mmol), paraformaldehyde (5 mmol), and sodium cyanoborohydride (1.5 mmol), acetic acid (10 mL) was added. The mixture was stirred at room temperature overnight and then poured into 100 mL of water. Saturated sodium bicarbonate was added to adjust the pH to 8–9. After standard workup with dichloromethane, the residue was purified by silica gel column chromatography using EtOAc/Hexane (1:5) to afford **3a** or **3b** as a white solid.

**3a:** (108 mg, 48%); ^1^H NMR (CD_3_CN): *δ* (ppm): 7.37 (d, 2H, *J* = 8.7 Hz), 7.34 (d, 2H, *J* = 8.8 Hz), 6.94 (d, 1H, *J* = 16.4 Hz), 6.96–6.93 (m, 1H), 6.90 (d, 1H, *J* = 16.4 Hz, 6.60 (d, 2H, *J* = 8.6 Hz), 2.79 (s, 3H).

**3b:** (60 mg, 50%); ^1^H NMR (CD_3_CN): *δ* (ppm): 7.40 (d, 2H, *J* = 5.2 Hz), 7.37 (d, 1H, *J* = 4.9 Hz), 6.93 (d, 3H, *J* = 1.9 Hz), 6.81 (d, 2H, *J* = 8.7 Hz), 6.76 (d, 2H, *J* = 8.9 Hz), 2.96 (s, 6H).

*Compound **4a** ((E)-4-(4-(dimethylamino)styryl)phenol) or **4b** ((E)-4-(4-(dimethylamino)styryl)-2-methoxyphenol).* To a mixture of compound **2a** or **2b** (5.0 mmol), paraformaldehyde (50 mmol), and sodium cyanoborohydride (24 mmol), acetic acid (100 mL) was added. The mixture was stirred at room temperature overnight and then poured into 100 mL of water. Saturated sodium bicarbonate was added to adjust the pH to 8–9. After standard workup with dichloromethane (3 × 50 mL), the residue was purified by silica gel column chromatography using EtOAc/Hexane (1:10) to afford **4a** or **4b** as a white solid.

**4a:** (60 mg, 50%); ^1^H NMR (CD_3_CN): *δ* (ppm): 7.40 (d, 2H, *J* = 5.2 Hz), 7.37 (d, 1H, *J* = 4.9 Hz), 6.93 (d, 3H, *J* = 1.9 Hz), 6.81 (d, 2H, *J* = 8.7 Hz), 6.76 (d, 2H, *J* = 8.9 Hz), 2.96 (s, 6H).

**4b:** (50 mg, 46%); ^1^H NMR (Acetone-d6): *δ* (ppm) 7.61 (s, 1H), 7.39 (d, 2H, *J* = 8.8 Hz), 7.18 (d, 1H, *J* = 2.0 Hz), 7.01–6.94 (m, 2H), 6.90 (d, 1H, *J* = 16.3 Hz), 6.79 (d, 1H, *J* = 8.1 Hz), 6.73 (d, 2H, *J* = 8.9 Hz), 3.89 (s, 3H), 2.96 (s, 6H).

#### 4.2.2. Synthesis of Compounds **L1**–**L12**

***L1****, (E)-2-((4,7-dimethyl-1,4,7-triazonan-1-yl)methyl)-4-(4-(dimethylamino)styryl)-6-methoxyphenol*. Paraformaldehyde (5 mg, 0.1 mmol) was added to a solution of 1,4-dimethyl-1,4,7-triazacyclononane (18 mg, 0.1 mmol) in MeCN (20 mL), and the resultant mixture was heated to reflux for 30 min. A solution of compound **4a** (27 mg, 0.1 mmol) in MeCN (10 mL) was added to the reaction flask and the solution was refluxed for an additional 24 h. The solvent was removed, and the resulting residue was purified by CombiFlash (reversed-phase) using MeCN/H_2_O/TFA (gradient wash from 10:90:1 to 30:70:1) to yield a light-yellow solution. The solution was neutralized by saturated NaHCO_3_ solution (30 mL) and extracted by dichloromethane (3 × 10 mL). The solvent was removed to yield a yellow semi-solid (13 mg, yield 30%). ^1^H NMR (CD_3_CN): δ (ppm): 7.39 (d, 2H, *J* = 8.8 Hz), 7.05 (d, 1H, *J* = 2.0 Hz), 6.95 (d, 1H, *J* = 16.4 Hz), 6.85 (d, 1H, *J* = 16.4 Hz), 6.79 (d, 1H, *J* = 2.0 Hz), 6.79 (d, 2H, *J* = 2.0 Hz), 3.86 (s, 3H), 3.83 (s, 2H), 2.95 (s, 6H), 2.91–2.80 (m, 4H), 2.67–2.60 (m, 4H), 2.55 (s, 4H), 2.36 (s, 6H). MS (ESI): Expected *m*/*z* 438.3068, found 438.3057 [M + H].

***L2****, (E)-2-((4,7-dimethyl-1,4,7-triazonan-1-yl)methyl)-6-methoxy-4-(4-(methylamino)styryl)phenol.* Paraformaldehyde (5 mg, 0.1 mmol) was added to a solution of 1,4-dimethyl-1,4,7-triazacyclononane (18 mg, 0.1 mmol) in MeCN (20 mL), and the resultant mixture was heated to reflux for 30 min. A solution of compound **3b** (25 mg, 0.1 mmol) in MeCN (10 mL) was added to the reaction flask, and the solution was refluxed for an additional 24 h. The solvent was removed, and the resulting residue was purified by CombiFlash (reversed-phase) using MeCN/H_2_O/TFA (gradient wash from 10:90:1 to 30:70:1) to yield a light-yellow solution. The solution was neutralized by saturated NaHCO_3_ solution (30 mL) and extracted by dichloromethane (3 × 10 mL). The solvent was removed to yield a yellow semi-solid (15 mg, yield 34%). ^1^H NMR (CDCl_3_): *δ* (ppm): 7.33 (d, 2H, *J* = 8.6 Hz), 6.95 (d, 1H, *J* = 1.9 Hz), 6.86 (d, 1H, *J* = 16.3 Hz), 6.78 (d, 1H, *J* = 16.3 Hz), 6.74 (d, 1H, *J* = 1.9 Hz), 6.59 (d, 1H, *J* = 8.6 Hz), 3.92 (s, 3H), 3.83 (s, 2H), 3.00–2.91 (m, 4H), 2.86 (s, 3H), 2.78–2.70 (m, 4H), 2.63 (s, 4H), 2.40 (s, 6H). MS (ESI): Expected *m*/*z* 424.2911, found 424.2901 [M + H].

***L3****, (E)-2-((4,7-dimethyl-1,4,7-triazonan-1-yl)methyl)-4-(4-(methylamino)styryl)phenol.* Paraformaldehyde (5 mg, 0.1 mmol) was added to a solution of 1,4-dimethyl-1,4,7-triazacyclononane (18 mg, 0.1 mmol) in MeCN (20 mL), and the resultant mixture was heated to reflux for 30 min. A solution of compound **3a** (22 mg, 0.1 mmol) in MeCN (10 mL) was added to the reaction flask, and the solution was refluxed for an additional 24 h. The solvent was removed, and the resulting residue was purified by CombiFlash (reversed-phase) using MeCN/H_2_O/TFA (gradient wash from 10:90:1 to 30:70:1) to yield a light-yellow solution. The solution was neutralized by saturated NaHCO_3_ solution (30 mL) and extracted by dichloromethane (3 × 10 mL). The solvent was removed to yield a yellow semi-solid (15 mg, 38%). ^1^H NMR (CD_3_CN): *δ* (ppm): 7.32 (d, 2H, *J* = 8.6 Hz), 7.28 (dd, 1H, *J* = 8.3, 2.3 Hz), 7.21 (d, 1H, *J* = 2.2 Hz), 6.91 (d, 1H, *J* = 16.4 Hz), 6.83 (d, 1H, *J* = 16.4 Hz), 6.76 (d, 1H, *J* = 8.3 Hz), 6.59 (d, 2H, *J* = 8.6 Hz), 4.51 (s, 1H), 3.86 (s, 2H), 2.93–2.83 (m, 4H), 2.78 (d, *J* = 3.1 Hz, 3H), 2.69–2.61 (m, 4H), 2.58 (s, 4H), 2.38 (s, 6H). MS (ESI): Expected *m*/*z* 394.2811, found 394.2802 [M + H].

***L4****, (E)-2-((4,7-dimethyl-1,4,7-triazonan-1-yl)methyl)-4-(4-(dimethylamino)styryl)phenol.* Paraformaldehyde (5 mg, 0.1 mmol) was added to a solution of 1,4-dimethyl-1,4,7-triazacyclononane (18 mg, 0.1 mmol) in MeCN (20 mL), and the resultant mixture was heated to reflux for 30 min. A solution of compound **4a** (24 mg, 0.1 mmol) in MeCN (10 mL) was added to the reaction flask, and the solution was refluxed for an additional 24 h. The solvent was removed, and the resulting residue was purified by CombiFlash (reversed-phase) using MeCN/H_2_O/TFA (gradient wash from 10:90:1 to 30:70:1) to yield a light-yellow solution. The solution was neutralized by saturated NaHCO_3_ solution (30 mL) and extracted by dichloromethane (3 × 10 mL). The solvent was removed to yield a yellow semi-solid (13 mg, yield 32%). ^1^H NMR (CD_3_CN): *δ* (ppm): 7.38 (d, 2H, *J* = 8.7 Hz), 7.28 (dd, 1H, *J* = 8.3, 2.2 Hz), 7.18 (d, *J* = 2.2 Hz, 1H), 6.92 (d, 1H, *J* = 16.4 Hz), 6.86 (d, 1H, *J* = 16.4 Hz), 6.74 (d, 1H, *J* = 8.8 Hz), 6.71 (d, 1H, *J* = 8.1 Hz), 3.83 (s, 2H), 2.95 (s, 6H), 2.88–2.82 (m, 4H), 2.66–2.58 (m, 4H), 2.53 (s, 4H), 2.35 (s, 6H). MS (ESI): Expected *m*/*z* 409.2962, found 408.2950 [M + H].

***L5****, (E)-4-(4-(diethylamino)styryl)-2-((4,7-dimethyl-1,4,7-triazonan-1-yl)methyl)-6-methoxyphenol.* A mixture of 4-vinylphenol (200 mg, 1.7 mmol), 4-bromo-*N,N-*diethylaniline (388 mg, 1.7 mmol), triethanolamine (10 mL), and Pd(OAc)_2_ (23 mg, 0.1 mmol) was stirred under nitrogen at 100 °C for 24 h. The reaction was cooled to room temperature and quenched with water (5 mL). The resulting solution was extracted by ethyl acetate (3 × 20 mL). The solvent was removed and purified by silica gel column chromatography (ethyl acetate: hexane = 1:4) to obtain crude product **5b**. Then, paraformaldehyde (6 mg, 0.2 mmol) was added to a solution of 1,4-dimethyl-1,4,7-triazacyclononane (15 mg, 0.1 mmol) in MeCN (10 mL), and the resultant mixture was heated to reflux for 30 min. A solution of compound **5b** (27 mg, 0.1 mmol) in MeCN (10 mL) was added to the reaction flask, and the solution was refluxed for an additional 24 h. The solvent was removed, and the resulting residue was purified by CombiFlash (reversed-phase) using MeCN/H_2_O/TFA (gradient wash from 10:90:1 to 30:70:1). The solution was neutralized by saturated NaHCO_3_ solution (30 mL), extracted by dichloromethane, and dried to give a yellow semi-solid (15 mg, yield 34%). ^1^H NMR (CDCl_3_): *δ* (ppm): 7.34 (d, 2H, *J* = 8.8 Hz), 7.27 (dd, 1H, *J* = 7.9 Hz), 7.11 (d, 1H, *J* = 2.2 Hz), 6.93–6.73 (m, 3H), 6.65 (d, 2H, *J* = 8.9 Hz), 3.83 (s, 2H), 3.37 (q, 4H, *J* = 7.1 Hz), 3.04–2.82 (m, 4H), 2.58 (s, 4H), 2.41 (s, 6H), 1.17 (t, *J* = 7.0 Hz, 6H). MS (ESI): Expected *m*/*z* 467.3381, found 467.3372 [M + H].

***L6****, (E)-4-(4-(diethylamino)styryl)-2-((4,7-dimethyl-1,4,7-triazonan-1-yl)methyl)phenol.* A mixture of 2-methoxy-4-vinylphenol (200 mg, 1.3 mmol), 4-bromo-*N,N*-diethylaniline (297 mg, 1.3 mmol), triethanolamine (10 mL), and Pd(OAc)_2_ (23 mg, 0.1 mmol) was stirred under nitrogen at 100 °C for 24 h. The reaction was cooled to room temperature and quenched with water (5 mL). The resulting solution was extracted by ethyl acetate (3 × 20 mL). The solvent was removed, and the product was purified by silica gel column chromatography (ethyl acetate: hexane = 1:4) to obtain crude material 5a. Then, paraformaldehyde (6 mg, 0.2 mmol) was added to a solution of 1,4-dimethyl-1,4,7-triazacyclononane (15 mg, 0.1 mmol) in MeCN (10 mL), and the resultant mixture was heated to reflux for 30 min. A solution of compound **5a** (20 mg, 0.05 mmol) in MeCN (10 mL) was added to the reaction flask, and the solution was refluxed for an additional 24 h. The solvent was removed, and the resulting residue was purified by CombiFlash (reversed-phase) using MeCN/H_2_O/TFA (gradient wash from 10:90:1 to 30:70:1). The solution was neutralized by saturated NaHCO_3_ solution (30 mL), extracted by dichloromethane (3 × 10 mL), and dried to give a yellow semi-solid (12 mg, yield 26%). ^1^H NMR (CDCl_3_): *δ* (ppm): 7.34 (d, 2H, *J* = 8.7 Hz), 6.95 (d, 1H, *J* = 1.8 Hz), 6.86 (d, 1H, *J* = 16.2 Hz), 6.81–6.72 (m, 2H), 6.65 (d, 2H, *J* = 8.8 Hz), 3.92 (s, 3H), 3.83 (s, 2H), 3.37 (q, 4H, *J* = 7.1 Hz), 2.99–2.86 (m, 4H), 2.80–2.70 (m, 4H), 2.63 (s, 4H), 2.40 (s, 6H), 1.17 (t, *J* = 7.0 Hz, 6H). MS (ESI): Expected *m*/*z* 437.3275, found 437.3261 [M + H].

***L7****, (E)-2-((4,7-dimethyl-1,4,7-triazonan-1-yl)methyl)-6-methoxy-4-(4-(piperidin-1-yl)styryl)phenol.* A mixture of 2-methoxy-4-vinylphenol (200 mg, 1.3 mmol), 1-(4-bromophenyl)piperidine (312 mg, 1.3 mmol), triethanolamine (10 mL), and Pd(OAc)_2_ (23 mg, 0.1 mmol) was stirred under nitrogen at 100 °C for 24 h. The reaction was cooled to room temperature and quenched with water (5 mL). The resulting solution was extracted by ethyl acetate (3 × 20 mL). The solvent was removed, and the product was purified by silica gel column chromatography (ethyl acetate: hexane = 1:4) to obtain crude material 7a. Then, paraformaldehyde (6 mg, 0.2 mmol) was added to a solution of 1,4-dimethyl-1,4,7-triazacyclononane (15 mg, 0.1 mmol) in MeCN (10 mL), and the resultant mixture was heated to reflux for 30 min. A solution of compound **7a** (20 mg, 0.05 mmol) in MeCN (10 mL) was added to the reaction flask, and the solution was refluxed for an additional 24 h. The solvent was removed, and the resulting residue was purified by CombiFlash (reversed-phase) using MeCN/H_2_O/TFA (gradient wash from 10:90:1 to 30:70:1). The solution was neutralized by saturated NaHCO_3_ solution (30 mL), extracted by dichloromethane (3 × 10 mL), and dried to give a yellow semi-solid (12 mg, yield 26%). ^1^H NMR (CDCl_3_): *δ* (ppm): 7.36 (d, 2H, *J* = 8.7 Hz), 6.95 (d, 1H, *J* = 1.9 Hz), 6.89 (d, 2H, *J* = 8.8 Hz), 6.84 (s, 2H), 6.74 (d, 1H, *J* = 1.9 Hz), 3.92 (s, 3H), 3.83 (s, 2H), 3.22–3.14 (m, 4H), 2.99–2.88 (m, 4H), 2.77–2.68 (m, 4H), 2.62 (s, 4H), 2.40 (s, 6H), 1.69 (m, 4H), 1.64–1.51 (m, 2H). MS (ESI): Expected *m*/*z* 479.3386, found 479.3375 [M + H].

***L8****, (E)-2-((4,7-dimethyl-1,4,7-triazonan-1-yl)methyl)-4-(4-(piperidin-1-yl)styryl)phenol.* A mixture of 4-vinylphenol (200 mg, 1.7 mmol), 1-(4-bromophenyl)piperidine (408 mg, 1.7 mmol), triethanolamine (10 mL) and Pd(OAc)_2_ (23 mg, 0.1 mmol) was stirred under nitrogen at 100 °C for 24 h. The reaction was cooled to room temperature and quenched with water (5 mL). The resulting solution was extracted by ethyl acetate (3 × 20 mL). The solvent was removed and purified by silica gel column chromatography (ethyl acetate: hexane = 1:4) to obtain a crude product **7b**. Then, paraformaldehyde (6 mg, 0.2 mmol) was added to a solution of 1,4-dimethyl-1,4,7-triazacyclononane (15 mg, 0.1 mmol) in MeCN (10 mL), and the resultant mixture was heated to reflux for 30 min. A solution of compound **7b** (27 mg, 0.1 mmol) in MeCN (10 mL) was added to the reaction flask, and the solution was refluxed for an additional 24 h. The solvent was removed, and the resulting residue was purified by CombiFlash (reversed-phase) using MeCN/H_2_O/TFA (gradient wash from 10:90:1 to 30:70:1). The solution was neutralized by saturated NaHCO_3_ solution (30 mL), extracted by dichloromethane, and dried to give a yellow semi-solid (15 mg, yield 34%). ^1^H NMR (CDCl_3_): *δ* (ppm): 7.36 (d, 2H, *J* = 8.8 Hz), 7.29 (dd, 1H, *J* = 8.4, 2.2 Hz), 7.11 (d, 1H, *J* = 2.2 Hz), 6.95–6.78 (m, 5H), 3.83 (s, 2H), 3.26–3.14 (m, 4H), 2.98–2.84 (m, 4H), 2.72–2.62 (m, 4H), 2.56 (s, 4H), 2.39 (s, 6H), 1.71 (m, 4H), 1.65–1.51 (m, 2H). MS (ESI): Expected *m*/*z* 449.3280, found 449.3262 [M + H].

***L9****, (E)-2-((4,7-dimethyl-1,4,7-triazonan-1-yl)methyl)-6-methoxy-4-(4-morpholinostyryl)phenol.* A mixture of 2-methoxy-4-vinylphenol (200 mg, 1.3 mmol), 4-(4-bromophenyl)morpholine (315 mg, 1.3 mmol), triethanolamine (10 mL), and Pd(OAc)_2_ (23 mg, 0.1 mmol) was stirred under nitrogen at 100 °C for 24 h. The reaction was cooled to room temperature and quenched with water (5 mL). The resulting solution was extracted by ethyl acetate (3 × 20 mL). The solvent was removed, and the product was purified by silica gel column chromatography (ethyl acetate: hexane = 1:4) to obtain crude material 7c. Then, paraformaldehyde (6 mg, 0.2 mmol) was added to a solution of 1,4-dimethyl-1,4,7-triazacyclononane (15 mg, 0.1 mmol) in MeCN (10 mL), and the resultant mixture was heated to reflux for 30 min. A solution of compound **7c** (20 mg, 0.05 mmol) in MeCN (10 mL) was added to the reaction flask, and the solution was refluxed for an additional 24 h. The solvent was removed, and the resulting residue was purified by CombiFlash (reversed-phase) using MeCN/H_2_O/TFA (gradient wash from 10:90:1 to 30:70:1). The solution was neutralized by saturated NaHCO_3_ solution (30 mL), extracted by dichloromethane (3 × 10 mL), and dried to give a yellow semi-solid (12 mg, yield 26%). ^1^H NMR (500 MHz, CDCl_3_) δ 7.33 (d, *J* = 8.7 Hz, 2H), 6.90 (s, 1H), 6.82 (d, *J* = 8.7 Hz, 2H), 6.80 (s, 2H), 6.72 (s, 1H), 3.86 (s, 3H), 3.79 (m, 8H), 3.15–3.09 (m, 4H), 2.86 (m, 4H), 2.76 (m, 4H), 2.40 (s, 6H). MS (ESI): Expected *m*/*z* 481.3179, found 481.3162 [M + H].

***L10****, (E)-2-((4,7-dimethyl-1,4,7-triazonan-1-yl)methyl)-4-(4-morpholinostyryl)phenol.* A mixture of 4-vinylphenol (200 mg, 1.7 mmol), 4-(4-bromophenyl)morpholine (411 mg, 1.7 mmol), triethanolamine (10 mL), and Pd(OAc)_2_ (23 mg, 0.1 mmol) was stirred under nitrogen at 100 °C for 24 h. The reaction was cooled to room temperature and quenched with water (5 mL). The resulting solution was extracted by ethyl acetate (3 × 20 mL). The solvent was removed and purified by silica gel column chromatography (ethyl acetate: hexane = 1:4) to obtain crude product **7d**. Then, paraformaldehyde (6 mg, 0.2 mmol) was added to a solution of 1,4-dimethyl-1,4,7-triazacyclononane (15 mg, 0.1 mmol) in MeCN (10 mL), and the resultant mixture was heated to reflux for 30 min. A solution of compound **7d** (27 mg, 0.1 mmol) in MeCN (10 mL) was added to the reaction flask, and the solution was refluxed for an additional 24 h. The solvent was removed, and the resulting residue was purified by CombiFlash (reversed-phase) using MeCN/H_2_O/TFA (gradient wash from 10:90:1 to 30:70:1). The solution was neutralized by saturated NaHCO_3_ solution (30 mL), extracted by dichloromethane, and dried to give a yellow semi-solid (15 mg, yield 34%). ^1^H NMR (CD_3_CN): *δ* (ppm): 7.42 (d, *J* = 8.8 Hz, 2H), 7.31 (dd, *J* = 8.3, 2.3 Hz, 1H), 7.23 (d, *J* = 2.2 Hz, 1H), 6.93 (d, *J* = 7.6 Hz, 4H), 6.75 (d, *J* = 8.3 Hz, 1H), 3.86 (s, 2H), 3.83–3.73 (m, 4H), 3.19–3.09 (m, 4H), 2.89–2.84 (m, 4H), 2.68–2.61 (m, 4H), 2.57 (s, 4H), 2.37 (s, 6H). MS (ESI): Expected *m*/*z* 450.3073, found 451.3059 [M + H].

***L11****, (E)-2-((4,7-dimethyl-1,4,7-triazonan-1-yl)methyl)-6-methoxy-4-(4-(4-methylpiperazin-1-yl)styryl)phenol.* A mixture of 2-methoxy-4-vinylphenol (200 mg, 1.3 mmol), 1-(4-bromophenyl)methylpiperazine (332 mg, 1.3 mmol), triethanolamine (10 mL), and Pd(OAc)_2_ (23 mg, 0.1 mmol) was stirred under nitrogen at 100 °C for 24 h. The reaction was cooled to room temperature and quenched with water (5 mL). The resulting solution was extracted by ethyl acetate (3 × 20 mL). The solvent was removed, and the product was purified by silica gel column chromatography (ethyl acetate: hexane = 1:4) to obtain crude material **7e**. Then, paraformaldehyde (6 mg, 0.2 mmol) was added to a solution of 1,4-dimethyl-1,4,7-triazacyclononane (15 mg, 0.1 mmol) in MeCN (10 mL), and the resultant mixture was heated to reflux for 30 min. A solution of compound **7e** (20 mg, 0.05 mmol) in MeCN (10 mL) was added to the reaction flask, and the solution was refluxed for an additional 24 h. The solvent was removed, and the resulting residue was purified by CombiFlash (reversed-phase) using MeCN/H_2_O/TFA (gradient wash from 10:90:1 to 30:70:1). The solution was neutralized by saturated NaHCO_3_ solution (30 mL), extracted by dichloromethane (3 × 10 mL), and dried to give a yellow semi-solid (12 mg, yield 26%). ^1^H NMR (CDCl_3_): *δ* (ppm) 7.41 (d, 2H, *J* = 8.7 Hz), 6.98 (d, 1H, *J* = 1.9 Hz), 6.93 (d, 2H, *J* = 10.9 Hz), 6.88 (s, 2H), 6.78 (d, 1H, *J* = 1.9 Hz), 3.95 (s, 3H), 3.86 (s, 2H), 3.29–3.24 (m, 4H), 3.02–2.92 (m, 4H), 2.81–2.73 (m, 4H), 2.68 (s, 4H), 2.63–2.58 (m, 4H), 2.44 (s, 6H), 2.38 (s, 3H). MS (ESI): Expected *m*/*z* 494.3495, found 494.3481 [M + H].

***L12****, (E)-2-((4,7-dimethyl-1,4,7-triazonan-1-yl)methyl)-4-(4-(4-methylpiperazin-1-yl)styryl)phenol.* A mixture of 4-vinylphenol (200 mg, 1.7 mmol), 1-(4-bromophenyl)methylpiperazine (433 mg, 1.7 mmol), triethanolamine (10 mL), and Pd(OAc)_2_ (23 mg, 0.1 mmol) was stirred under nitrogen at 100 °C for 24 h. The reaction was cooled to room temperature and quenched with water (5 mL). The resulting solution was extracted by ethyl acetate (3 × 20 mL). The solvent was removed and purified by silica gel column chromatography (ethyl acetate: hexane = 1:4) to obtain crude product 7f. Then, paraformaldehyde (6 mg, 0.2 mmol) was added to a solution of 1,4-dimethyl-1,4,7-triazacyclononane (15 mg, 0.1 mmol) in MeCN (10 mL), and the resultant mixture was heated to reflux for 30 min. A solution of compound **7f** (27 mg, 0.1 mmol) in MeCN (10 mL) was added to the reaction flask, and the solution was refluxed for an additional 24 h. The solvent was removed, and the resulting residue was purified by CombiFlash (reversed-phase) using MeCN/H_2_O/TFA (gradient wash from 10:90:1 to 30:70:1). The solution was neutralized by saturated NaHCO_3_ solution (30 mL), extracted by dichloromethane, and dried to give a yellow semi-solid (15 mg, yield 34%). ^1^H NMR (500 MHz, CDCl_3_) δ 7.30 (d, 2H, *J* = 8.9 Hz), 7.11 (s, 1H), 6.82 (d, 2H, *J* = 8.5 Hz), 6.79 (s, 4H), 3.76 (s, 2H), 3.17 (m, 4H), 2.83–2.80 (m, 4H), 2.70–2.73 (m, 4H), 2.63–2.59 (m, 4H), 2.54–2.47 (m, 4H), 2.35 (s, 6H), 2.28 (s, 3H). MS (ESI): Expected *m*/*z* 464.3389, found 464.3368 [M + H].

### 4.3. Fluorescence Spectra Measurements

All fluorescence measurements were performed using a SpectraMax M2e plate reader (Molecular Devices, San Jose, CA, USA). The fluorescence spectra of the compounds’ solution (100 μL PBS, 5.0 µM) were recorded with excitation at 380 nm and an emission wavelength from 400 to 680 nm. To PBS solutions (100 μL) of the compounds (5.0 µM), various Aβ_42_ species were added, with final concentrations of 5.0 µM and 25.0 µM, and their fluorescence spectra (λ_max_ of emission) were determined as described above.

### 4.4. Amyloid β Peptide Experiments

#### 4.4.1. Fluorescence Turn-On and Cell Studies

Aβ_42_ powder was prepared by dissolving commercial Aβ_42_ peptide (AnaSpec or GL Biochem) in ammonium hydroxide solution (1%, *v*/*v*). The solution was then aliquoted out and lyophilized overnight. The resulting aliquoted powder was stored at −80 °C. Aβ_42_ monomers were generated by dissolving Aβ_42_ powder in 1,1,1,3,3,3-hexafluoro-2-propanol (HFIP, 1 mM) and incubating for 1 h at room temperature. The solution was then evaporated overnight and dried by vacuum centrifuge to result in monomeric films. Aβ_42_ fibrils were generated by dissolving the monomeric Aβ_42_ films in DMSO, diluting into the appropriate buffer, and incubating for 24 h at 37 °C with continuous agitation (final DMSO concentration was <2%). For the preparation of Aβ_42_ oligomers, the peptides were suspended in PBS buffer and incubated overnight at 4 °C. (The ThT-negative control, since ThT does not bind to soluble aggregates [56], and UV-Vis confirming that sAβ_42_, but not fibrillar Aβ_42_, was formed are shown in the Supplementary Materials Figure S3) Amyloid beta procedures were validated in previous publications [16,35].

#### 4.4.2. Inhibition Assay

For monitoring the inhibition of Aβ_40_ fibrils, a peptide solution of 25 μM Aβ_40_ monomers (AnaSpec, Fremont, CA, USA) was prepared and incubated at 37 °C with shaking for 48 h (final DMSO concentration was <2%), in the presence of 5 μM ThT and 5 μM of compound. The ThT fluorescence signal was monitored at 485 nm upon 435 nm excitation. This procedure was established in a previous publication [57].

### 4.5. Alamar Blue Assay

Mouse neuroblastoma Neuro2A (N2A) cell lines were purchased from the American Type Culture Collection (ATCC), Manassas, VA, USA. Cells were grown in DMEM/10% FBS, which is the regular growth media for N2A cells, in a humidified atmosphere with 5% CO_2_ at 37 °C. N2A cells were seeded in a clear 96-well plate (2.5 × 10^4^/well) with DMEM/10% FBS. The media was changed to DMEM/N-2 media 24 h later. After 1 h, the treatment reagents were added. Owing to the poor solubility of compounds in the media, the final amount of DMSO used was 1% (*v*/*v*). After an additional incubation of 40 h, Alamar Blue solution was added to each well, and the cells were incubated for 90 min at 37 °C. Fluorescence intensity was measured at 590 nm (excitation wavelength = 560 nm).

#### 4.5.1. Cytotoxicity Studies

The Alamar Blue assay was carried out as described, with the following treatment reagents: 20 μM compounds, 10 μM compounds, 5 μM compounds, and 2 μM compounds.

#### 4.5.2. N2a Cell Rescue Studies

The Alamar Blue assay was carried out as described, with the following treatment reagents: 20 μM Aβ_42_ species, 5 μM compounds, and 20 μM CuCl_2_.

### 4.6. Histological Staining of 5xFAD Mice Brain Sections

To evaluate the Aβ binding specificity of the compounds in vitro, brain sections from 11-month-old 5xFAD mice were washed with PBS (3 × 5 min) and blocked with bovine serum albumin (2% *w*/*v* BSA in PBS, pH 7.4, 10 min). Then, the sections were incubated with 25 μM of multifunctional compounds and sequentially stained with HJ3.4 antibody (Professor David Holtzman, 1 μg/mL) for 30 min. The brain sections were treated with 2% *w*/*v* BSA-PBS again for 4 min to remove any compounds or antibodies that were non-specifically binding to the tissue. Finally, the sections were washed with PBS (3 × 2 min) and DI water (2 min) and mounted with non-fluorescent mounting media (Vectashield). Colocalization analysis and determination of Pearson’s correlation coefficient were performed with the imaging software Fiji (ImageJ 1.52p). Mouse brains were harvested in compliance with the Institutional Animal Care and Use Committee of the University of Illinois at Urbana-Champaign (IACUC protocol #22094).

### 4.7. Molecular Docking

Molecular docking studies were performed with the Schrödinger Suite software Maestro 14.2. Protein structure Aβ_42_ tetramers (PDB ID: 6RHY) and the Aβ_42_ fibrils (PDB ID: 5OQV) were imported from the RCSB database and optimized by minimal minimization with the OPLS3 force field using the Protein Preparation Wizard program. The LS stilbene derivatives were prepared using Ligprep, and the pH was set to 7.0 ± 2.0 using Epik. The resulting different protonation states of the compounds were obtained and used for docking studies. The grid size was set to include the whole optimized protein structure in each direction. Final molecular docking was performed using Glide. The calculated poses were ranked by both docking score and Glide e-model energy, and the structures with the best docking scores and Glide e-model energies were rendered in PyMol 3.1.

### 4.8. Log D Measurement

The determination of log D values for N-alkylamino multifunctional compounds was performed using a slightly modified reported procedure [58]. The compounds (final concentration 10 μM) were added to the premix suspension containing 0.5 mL octanol and 0.5 mL octanol-saturated PBS buffer (pH = 7.4). The resulting mixture was stirred vigorously for 5 min and centrifuged at 2000 rpm for 5 min. The octanol layer was separated from the PBS layer, and the fluorescence spectrum was recorded. The remaining 0.5 mL PBS layer was partitioned with 0.5 mL PBS-saturated octanol, and the octanol layer was separated after 5 min of vigorous shaking and centrifuging. The fluorescence spectrum of the octanol layer was recorded. The log D value was calculated based on the fluorescence intensity ratio for the two octanol layers. Each experiment was completed in triplicate, and the error represents the standard deviation for the average log D values.

## 5. Conclusions

Aβ oligomer-targeting dyes are highly sought after for both fundamental and translational research applications. The series of compounds described herein can serve as chemical tools, such as aggregation inhibitors or amyloid handles, for the development of better and more sAβ-selective compounds. By understanding the electronic contributions of EDGs and EAGs on a stilbene π system, the effect of these groups in cellular toxicity, and their participation in amyloid binding, we have established that these compounds can be effective in the appropriate amyloid-based assay.

O-methoxy-containing compounds generally have decreased amyloid specificity, reduced toxicity, the ability to rescue Cu-induced Aβ_42_ toxicity, and lower overall fluorescence compared to the non-methoxy analogs. Based on the docking studies, the different binding modes of these compounds to Aβ fibril and oligomer models suggest that the slight electronic contributions of EDGs/EAGs to TICT can be inferred from the fluorescence assays, as the fluorescence turn-ons between Aβ fibrils and soluble aggregates were similar. Hence, the fluorescence intensities and turn-ons show that TICT fluorescence depends on the EDG–EAG pair rather than the individual strength of electron donation and/or acceptance of each group. Another insight from molecular docking was that for sAβ, turn-on preference amphiphilicity is likely not solely responsible, as previously suggested [23]. The length and planarity of the hydrophobic stilbene ligand may also be important. Despite several of these compounds having incompatible toxicity profiles for cellular studies, compounds such as **L4** and **L8** showed promise by halting aggregation before the development of insoluble fibrils, having promising toxicity profiles for use as leads in the further development of molecular tools.

**Figure 8:**
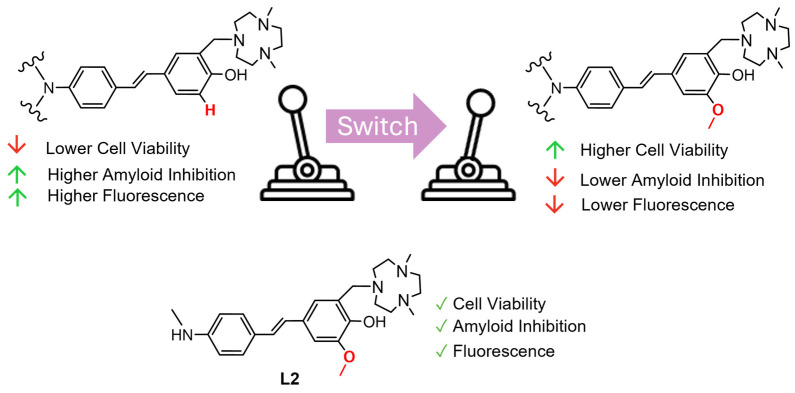
A summary of the structure-activity relationships determined by this series of N-alkylamino stilbene compounds. **L2** is the most promising Aβ inhibitor for use in assays.

Current Aβ dyes have high affinity but suffer low selectivity for specific amyloid species: soluble and insoluble. Small molecule inhibitors with both high affinity and selectivity to certain Aβ species would be immensely useful for both fundamental and translational research applications. The compounds reported herein are inhibitors of amyloid aggregation and can be used to modulate the formation of aggregates in vitro. **L2**, as shown in summarizing Figure 8, would be the best inhibitor for Aβ aggregation, based on its low toxicity, ample inhibition, and fluorescence properties. Overall, we present these compounds as chemical tools for use in assays for the development of necessary Aβ oligomer-targeting dyes.

## Data Availability

The experimental data used to support the results of this study are available in the article and in the Supplementary Materials.

## Supplementary Materials

The following supporting information can be downloaded at: https://www.mdpi.com/article/10.3390/molecules30112471/s1 and contains fluorescence spectra, molecular docking results, inhibition assay data, HPLC Chromatograms, absorbance spectra, soluble amyloid beta control data, and logD measurements are also included [59,60,61].

## Abbreviations

The following abbreviations are used in this manuscript:

AD	Alzheimer’s disease
Aβ	Amyloid beta
CR	Congo Red
EAG	Electron accepting group
EDG	Electron donating group
NMR	Nuclear magnetic resonance
sAβ	Soluble amyloid beta
tacn	1,4,7-triazacyclononane
ThT	Thioflavin T
TICT	Twisted Intramolecular Charge Transfer

## References

[1] Liu P.P., Xie Y., Meng X.Y., Kang J.S. (2019). History and progress of hypotheses and clinical trials for Alzheimer’s disease. Signal Transduct. Target. Ther..

[2] Ono K., Condron M.M., Teplow D.B. (2009). Structure–neurotoxicity relationships of amyloid β-protein oligomers. Proc. Natl. Acad. Sci. USA.

[3] Tew D.J., Bottomley S.P., Smith D.P., Ciccotosto G.D., Babon J., Hinds M.G., Masters C.L., Cappai R., Barnham K.J. (2008). Stabilization of Neurotoxic Soluble β-Sheet-Rich Conformations of the Alzheimer’s Disease Amyloid-β Peptide. Biophys. J..

[4] Jin L., Wu W.-H., Li Q.-Y., Zhao Y.-F., Li Y.-M. (2011). Copper inducing Aβ42 rather than Aβ40 nanoscale oligomer formation is the key process for Aβ neurotoxicity. Nanoscale.

[5] Yang J., Zeng F., Li X., Ran C., Xu Y., Li Y. (2020). Highly Specific Detection of Aβ Oligomers in Early Alzheimer’s Disease by a Near-Infrared Fluorescent Probe with a “V-shaped” Spatial Conformation. Chem. Commun..

[6] Matsumura K., Ono M., Kitada A., Watanabe H., Yoshimura M., Iikuni S., Kimura H., Okamoto Y., Ihara M., Saji H. (2015). Structure–Activity Relationship Study of Heterocyclic Phenylethenyl and Pyridinylethenyl Derivatives as Tau-Imaging Agents That Selectively Detect Neurofibrillary Tangles in Alzheimer’s Disease Brains. J. Med. Chem..

[7] Leuzy A., Chiotis K., Lemoine L., Gillberg P.-G., Almkvist O., Rodriguez-Vieitez E., Nordberg A. (2019). Tau PET imaging in neurodegenerative tauopathies—Still a challenge. Mol. Psychiatry.

[8] Vadukul D.M., Maina M., Franklin H., Nardecchia A., Serpell L.C., Marshall K.E. (2020). Internalisation and toxicity of amyloid-beta 1–42 are influenced by its conformation and assembly state rather than size. FEBS Lett..

[9] Tolar M., Hey J., Power A., Abushakra S. (2021). Neurotoxic Soluble Amyloid Oligomers Drive Alzheimer’s Pathogenesis and Represent a Clinically Validated Target for Slowing Disease Progression. Int. J. Mol. Sci..

[10] Ciaglia T., Miranda M.R., Di Micco S., Vietri M., Smaldone G., Musella S., Di Sarno V., Auriemma G., Sardo C., Moltedo O. (2024). Neuroprotective Potential of Indole-Based Compounds: A Biochemical Study on Antioxidant Properties and Amyloid Disaggregation in Neuroblastoma Cells. Antioxidants.

[11] Wang Q., Dong X., Zhang R., Zhao C. (2021). Flavonoids with Potential Anti-Amyloidogenic Effects as Therapeutic Drugs for Treating Alzheimer’s Disease. J. Alzheimer’s Dis..

[12] Radbakhsh S., Barreto G.E., Bland A.R., Sahebkar A. (2021). Curcumin: A small molecule with big functionality against amyloid aggregation in neurodegenerative diseases and type 2 diabetes. BioFactors.

[13] Hilt S., Liu R., Maezawa I., Rojalin T., Aung H.H., Budamagunta M., Slez R., Gong Q., Carney R.P., Voss J.C. (2022). Novel Stilbene-Nitroxyl Hybrid Compounds Display Discrete Modulation of Amyloid Beta Toxicity and Structure. Front. Chem..

[14] Arbo B.D., André-Miral C., Nasre-Nasser R.G., Schimith L.E., Santos M.G., Costa-Silva D., Muccillo-Baisch A.L., Hort M.A. (2020). Resveratrol Derivatives as Potential Treatments for Alzheimer’s and Parkinson’s Disease. Front. Aging Neurosci..

[15] Yu Z., Guo W., Patel S., Cho H.-J., Sun L., Mirica L.M. (2022). Amphiphilic stilbene derivatives attenuate the neurotoxicity of soluble Aβ42 oligomers by controlling their interactions with cell membranes. Chem. Sci..

[16] Sun L., Cho H.-J., Sen S., Arango A.S., Huynh T.T., Huang Y., Bandara N., Rogers B.E., Tajkhorshid E., Mirica L.M. (2021). Amphiphilic Distyrylbenzene Derivatives as Potential Therapeutic and Imaging Agents for the Soluble Amyloid-β Oligomers in Alzheimer’s Disease. J. Am. Chem. Soc..

[17] Fu Z., Yang J., Wei Y., Li J. (2016). Effects of piceatannol and pterostilbene against β-amyloid-induced apoptosis on the PI3K/Akt/Bad signaling pathway in PC12 cells. Food Funct..

[18] Yuan W., Shang Z., Qiang X., Tan Z., Deng Y. (2014). Synthesis of pterostilbene and resveratrol carbamate derivatives as potential dual cholinesterase inhibitors and neuroprotective agents. Res. Chem. Intermed..

[19] Garcia G.X., Larsen S.W., Pye C., Galbreath M., Isovitsch R., Fradinger E.A. (2013). The functional group on (E)-4,4′–disubstituted stilbenes influences toxicity and antioxidative activity in differentiated PC-12 cells. Bioorg. Med. Chem. Lett..

[20] Breitung E.M., Shu C.-F., McMahon R.J. (2000). Thiazole and Thiophene Analogues of Donor−Acceptor Stilbenes:  Molecular Hyperpolarizabilities and Structure−Property Relationships. J. Am. Chem. Soc..

[21] Gao Y., Li J., Wu Q., Wang S., Yang S., Li X., Chen N., Li L., Zhang L. (2021). Tetrahydroxy stilbene glycoside ameliorates Alzheimer’s disease in APP/PS1 mice via glutathione peroxidase related ferroptosis. Int. Immunopharmacol..

[22] Freyssin A., Page G., Fauconneau B., Rioux Bilan A. (2020). Natural stilbenes effects in animal models of Alzheimer’s disease. Neural Reg. Res..

[23] Gao D., Hao J.-p., Li B.-y., Zheng C.-c., Miao B.-b., Zhang L., Li Y.-l., Li L., Li X.-j., Zhang L. (2023). Tetrahydroxy stilbene glycoside ameliorates neuroinflammation for Alzheimer’s disease via cGAS-STING. Eur. J. Pharmacol..

[24] Firdoos S., Dai R., Tahir R.A., Khan Z.Y., Li H., Zhang J., Ni J., Quan Z., Qing H. (2023). In silico identification of novel stilbenes analogs for potential multi-targeted drugs against Alzheimer’s disease. J. Mol. Mod..

[25] Yu Z., Moshood Y., Wozniak M.K., Patel S., Terpstra K., Llano D.A., Dobrucki L.W., Mirica L.M. (2023). Amphiphilic Molecules Exhibiting Zwitterionic Excited-State Intramolecular Proton Transfer and Near-Infrared Emission for the Detection of Amyloid β Aggregates in Alzheimer’s Disease. Chem. Eur. J..

[26] Cho H.-J., Huynh T.T., Rogers B.E., Mirica L.M. (2020). Design of a multivalent bifunctional chelator for diagnostic ^64^Cu PET imaging in Alzheimer’s disease. Proc. Natl. Acad. Sci. USA.

[27] Cui M., Ono M., Watanabe H., Kimura H., Liu B., Saji H. (2014). Smart Near-Infrared Fluorescence Probes with Donor–Acceptor Structure for in Vivo Detection of β-Amyloid Deposits. J. Am. Chem. Soc..

[28] Kung H.F., Choi S.R., Qu W., Zhang W., Skovronsky D. (2010). ^18^F Stilbenes and Styrylpyridines for PET Imaging of Aβ Plaques in Alzheimer’s Disease: A Miniperspective. J. Med. Chem..

[29] Martí-Centelles R., Falomir E., Murga J., Carda M., Marco J.A. (2015). Inhibitory effect of cytotoxic stilbenes related to resveratrol on the expression of the VEGF, hTERT and c-Myc genes. Eur. J. Med. Chem..

[30] Kung H.F., Kung M.-P., Zhuang Z.-P. (2005). Stilbene Derivatives and Their Use for Binding and Imaging Amyloid Plaques. International Patent.

[31] Xiao G., Li Y., Qiang X., Xu R., Zheng Y., Cao Z., Luo L., Yang X., Sang Z., Su F. (2017). Design, synthesis and biological evaluation of 4′-aminochalcone-rivastigmine hybrids as multifunctional agents for the treatment of Alzheimer’s disease. Bioorg. Med. Chem..

[32] Lennol M.P., Canelles S., Guerra-Cantera S., Argente J., García-Segura L.M., de Ceballos M.L., Chowen J.A., Frago L.M. (2021). Amyloid-β1-40 differentially stimulates proliferation, activation of oxidative stress and inflammatory responses in male and female hippocampal astrocyte cultures. Mech. Ageing Dev..

[33] Gu L., Guo Z. (2013). Alzheimer’s Aβ42 and Aβ40 peptides form interlaced amyloid fibrils. J. Neurochem..

[34] Meisl G., Yang X., Hellstrand E., Frohm B., Kirkegaard J.B., Cohen S.I.A., Dobson C.M., Linse S., Knowles T.P.J. (2014). Differences in nucleation behavior underlie the contrasting aggregation kinetics of the Aβ40 and Aβ42 peptides. Proc. Natl. Acad. Sci. USA.

[35] Sharma A.K., Pavlova S.T., Kim J., Kim J., Mirica L.M. (2013). The effect of Cu^2+^ and Zn^2+^ on the Aβ_42_ peptide aggregation and cellular toxicity. Metallomics.

[36] Sun L., Sharma A.K., Han B.-H., Mirica L.M. (2020). Amentoflavone: A Bifunctional Metal Chelator that Controls the Formation of Neurotoxic Soluble Aβ42 Oligomers. ACS Chem. Neurosci..

[37] Cho H.-J., Sharma A.K., Zhang Y., Gross M.L., Mirica L.M. (2020). A Multifunctional Chemical Agent as an Attenuator of Amyloid Burden and Neuroinflammation in Alzheimer’s Disease. ACS Chem. Neurosci..

[38] Raju S.K., Sundhararajan N., Sekar P., Nagalingam Y. (2023). Therapeutic aspects of biologically potent vanillin derivatives: A critical review. J. Drug Deliv. Therap..

[39] Iannuzzi C., Liccardo M., Sirangelo I. (2023). Overview of the Role of Vanillin in Neurodegenerative Diseases and Neuropathophysiological Conditions. Int. J. Mol. Sci..

[40] Kumar S.N., Nair H.R., Kumar B.P. (2023). Comparative analysis of anti-oxidant potential of vanillin and ferulic acid invitro. Food Humanit..

[41] Terpstra K., Wang Y., Huynh T.T., Bandara N., Cho H.-J., Rogers B.E., Mirica L.M. (2022). Divalent 2-(4-Hydroxyphenyl)benzothiazole Bifunctional Chelators for 64Cu Positron Emission Tomography Imaging in Alzheimer’s Disease. Inorg. Chem..

[42] Liao F., Li A., Xiong M., Bien-Ly N., Jiang H., Zhang Y., Finn M.B., Hoyle R., Keyser J., Lefton K.B. (2018). Targeting of nonlipidated, aggregated apoE with antibodies inhibits amyloid accumulation. J. Clin. Investig..

[43] Mahan T.E., Wang C., Bao X., Choudhury A., Ulrich J.D., Holtzman D.M. (2022). Selective reduction of astrocyte apoE3 and apoE4 strongly reduces Aβ accumulation and plaque-related pathology in a mouse model of amyloidosis. Mol. Neurodegener..

[44] Esparza T.J., Zhao H., Cirrito J.R., Cairns N.J., Bateman R.J., Holtzman D.M., Brody D.L. (2013). Amyloid-beta oligomerization in Alzheimer dementia versus high-pathology controls. Ann. Neurol..

[45] Gremer L., Schölzel D., Schenk C., Reinartz E., Labahn J., Ravelli R.B.G., Tusche M., Lopez-Iglesias C., Hoyer W., Heise H. (2017). Fibril structure of amyloid-β(1–42) by cryo–electron microscopy. Science.

[46] Zou Y., Qian Z., Chen Y., Qian H., Wei G., Zhang Q. (2019). Norepinephrine Inhibits Alzheimer’s Amyloid-β Peptide Aggregation and Destabilizes Amyloid-β Protofibrils: A Molecular Dynamics Simulation Study. ACS Chem. Neurosci..

[47] Gautieri A., Beeg M., Gobbi M., Rigoldi F., Colombo L., Salmona M. (2019). The Anti-Amyloidogenic Action of Doxycycline: A Molecular Dynamics Study on the Interaction with Aβ42. Int. J. Mol. Sci..

[48] Ciudad S., Puig E., Botzanowski T., Meigooni M., Arango A.S., Do J., Mayzel M., Bayoumi M., Chaignepain S., Maglia G. (2020). Aβ(1-42) tetramer and octamer structures reveal edge conductivity pores as a mechanism for membrane damage. Nat. Commun..

[49] Lu C., Wu C., Ghoreishi D., Chen W., Wang L., Damm W., Ross G.A., Dahlgren M.K., Russell E., Von Bargen C.D. (2021). OPLS4: Improving Force Field Accuracy on Challenging Regimes of Chemical Space. J. Chem. Theory Comput..

[50] Friesner R.A., Murphy R.B., Repasky M.P., Frye L.L., Greenwood J.R., Halgren T.A., Sanschagrin P.C., Mainz D.T. (2006). Extra Precision Glide:  Docking and Scoring Incorporating a Model of Hydrophobic Enclosure for Protein−Ligand Complexes. J. Med. Chem..

[51] Friesner R.A., Banks J.L., Murphy R.B., Halgren T.A., Klicic J.J., Mainz D.T., Repasky M.P., Knoll E.H., Shelley M., Perry J.K. (2004). Glide:  A New Approach for Rapid, Accurate Docking and Scoring. 1. Method and Assessment of Docking Accuracy. J. Med. Chem..

[52] Dong X., Qiao Q., Qian Z., Wei G. (2018). Recent computational studies of membrane interaction and disruption of human islet amyloid polypeptide: Monomers, oligomers and protofibrils. Biochim. Biophys. Acta-Biomembr..

[53] Gao D., Wan J., Zou Y., Gong Y., Dong X., Xu Z., Tang J., Wei G., Zhang Q. (2022). The destructive mechanism of Aβ1–42 protofibrils by norepinephrine revealed via molecular dynamics simulations. Phys. Chem. Chem. Phys..

[54] Chen Z.-L., Singh P.K., Calvano M., Norris E.H., Strickland S. (2023). A possible mechanism for the enhanced toxicity of beta-amyloid protofibrils in Alzheimer’s disease. Proc. Natl. Acad. Sci. USA.

[55] Vangala V.R., Bhogala B.R., Dey A., Desiraju G.R., Broder C.K., Smith P.S., Mondal R., Howard J.A.K., Wilson C.C. (2003). Correspondence between Molecular Functionality and Crystal Structures. Supramolecular Chemistry of a Family of Homologated Aminophenols. J. Am. Chem. Soc..

[56] Jameson L.P., Smith N.W., Dzyuba S.V. (2012). Dye-Binding Assays for Evaluation of the Effects of Small Molecule Inhibitors on Amyloid (Aβ) Self-Assembly. ACS Chem. Neurosci..

[57] Terpstra K., Huang Y., Na H., Sun L., Gutierrez C., Yu Z., Mirica L.M. (2024). 2-Phenylbenzothiazolyl iridium complexes as inhibitors and probes of amyloid β aggregation. Dalton Trans..

[58] Ran C., Xu X., Raymond S.B., Ferrara B.J., Neal K., Bacskai B.J., Medarova Z., Moore A. (2009). Design, Synthesis, and Testing of Difluoroboron-Derivatized Curcumins as Near-Infrared Probes for in Vivo Detection of Amyloid-β Deposits. J. Am. Chem. Soc..

[59] Xue C., Lee Y.K., Tran J., Chang D., Guo Z. (2017). A mix-and-click method to measure amyloid-β concentration with sub-micromolar sensitivity. R. Soc. Open Sci..

[60] Groenning M. (2010). Binding mode of Thioflavin T and other molecular probes in the context of amyloid fibrils—Current status. J. Chem. Biol..

[61] Ertl P. (2022). A Web Tool for Calculating Substituent Descriptors Compatible with Hammett Sigma Constants. Chem. Methods.

