# Supplementary material for: N-Alkylamino Stilbene Compounds as Amyloid β Inhibitors for Alzheimer’s Disease Research
Source: Molecules. 2025 Jun 5;30(11):2471. doi: 10.3390/molecules30112471 (PMC12156033; doi:10.3390/molecules30112471)
Supplement: Supplementary file 1 [file molecules-30-02471-s001.zip › molecules-3622710-supplementary.pdf]

SUPPORTING INFORMATION

for

N-Alkylamino Stilbene Compounds as Amyloid β Inhibitors for Alzheimer's Disease Research

Citlali Gutiérrez[1], Liang Sun,[1] Yiran Huang,[1] Karna Terpstra,[1] Kai Gui,[1] and Liviu M. Mirica[1,2,*]

[1] Department of Chemistry, 600 S. Matthews Avenue, Urbana, Illinois 61801, United States

[2] Beckman Institute for Advanced Science and Technology, Carle Illinois College of Medicine, The Neuroscience Program, Department of Bioengineering, Carle Woese Institute for Genomic Biology, University of Illinois Urbana-Champaign, Urbana, Illinois, 61801, United States

*e-mail: mirica@illinois.edu.

Table of Contents **Page No.**

1. HPLC Chromatograms S2
2. Absorbance Spectra S5
3. Fluorescence Spectra S7
4. Aβ Inhibition Assays S9
5. ThT Control Studies S10
6. Molecular Docking Data S11
7. Log D Measurements S12
8. Hammett Approximations S13
9. Molecular Docking Images S15
10. References S19

1. HPLC Chromatograms

L1: VWD Wavelength = 230 nm

Purity: 80%

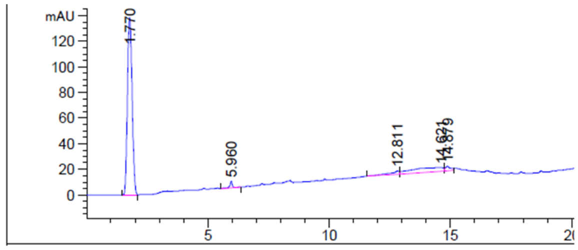

L2: VWD Wavelength = 230 nm

Purity: 96%

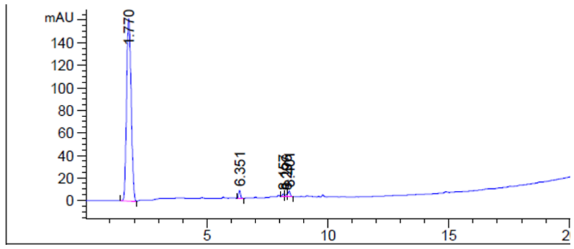

L3: VWD Wavelength = 230 nm

Purity: 74%

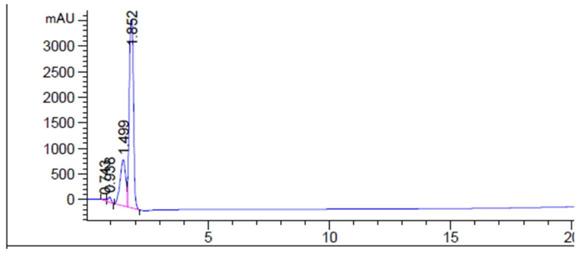

L4: VWD Wavelength = 260 nm

Purity: 85%

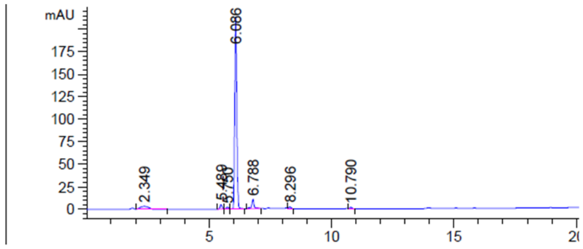

L5: VWD Wavelength = 260 nm

Purity: 79%

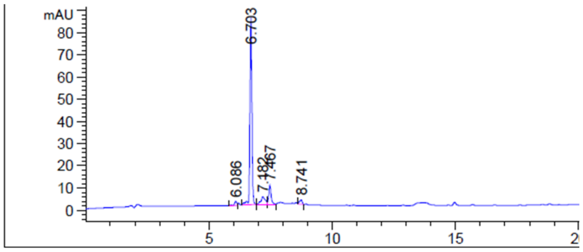

L6: VWD Wavelength = 260 nm

Purity: 94%

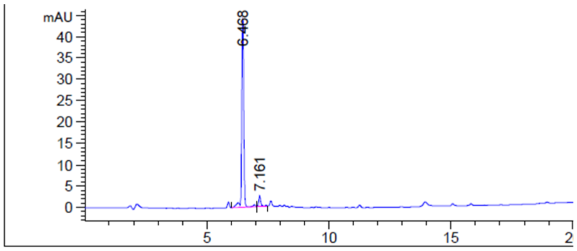

L7: VWD Wavelength = 260 nm

Purity: 100 %

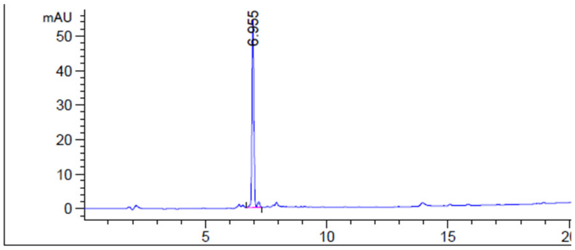

L8: VWD Wavelength = 260 nm

Purity: 90%

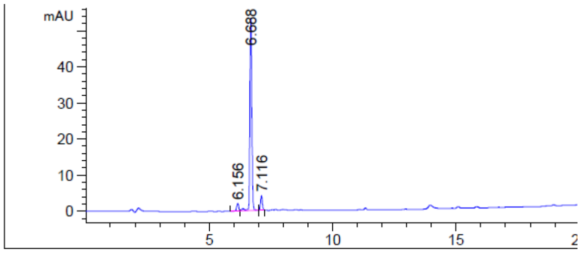

L9: VWD Wavelength = 260 nm

Purity: 90%

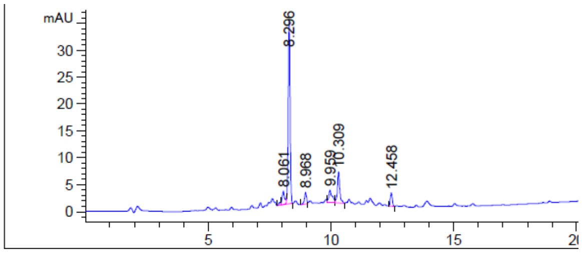

L10: VWD Wavelength = 260 nm

Purity: 84%

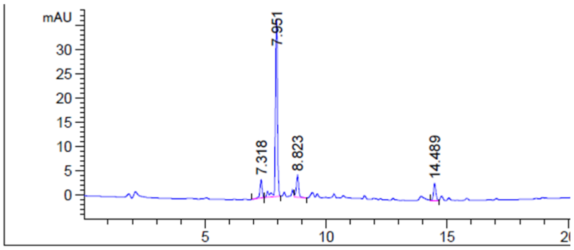

2. Absorbance Spectra

L1

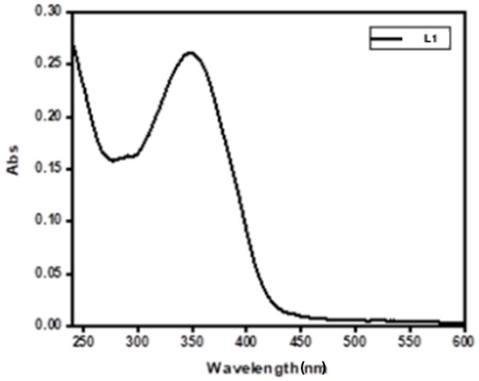

L2

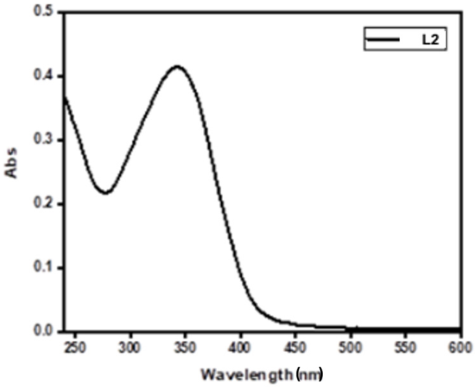

L3

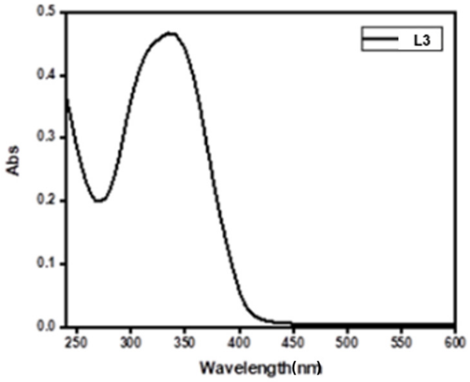

L4

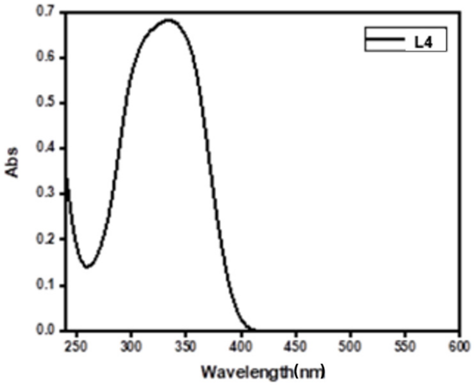

L5

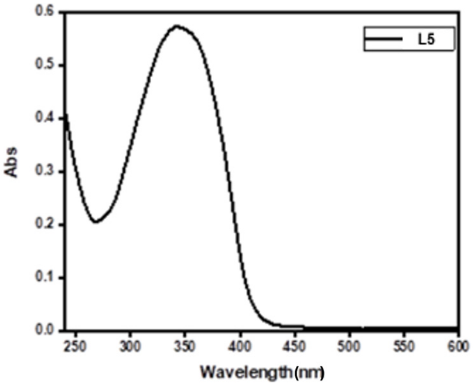

L6

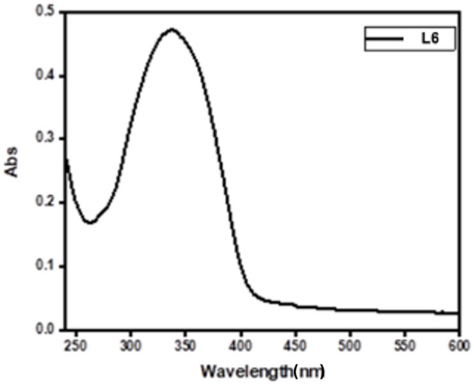

L7

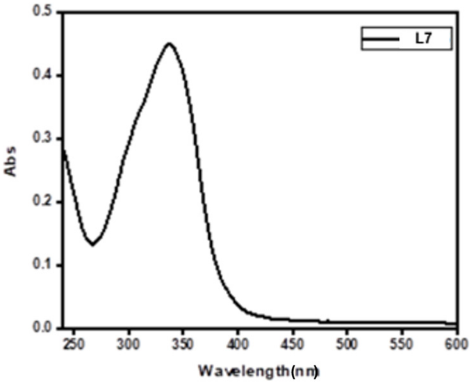

L8

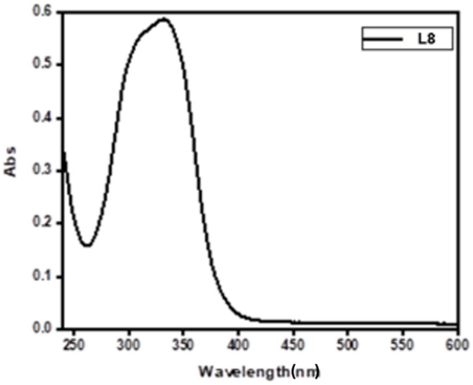

L9

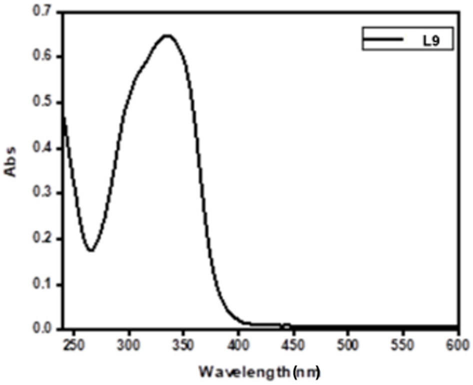

L10

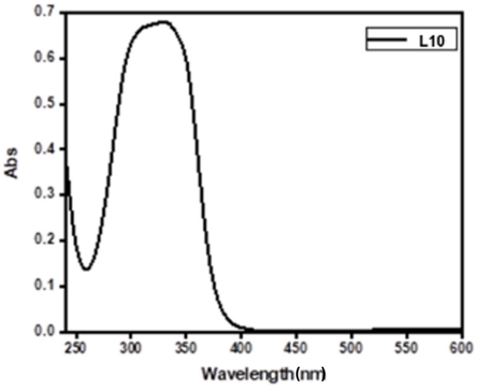

L11

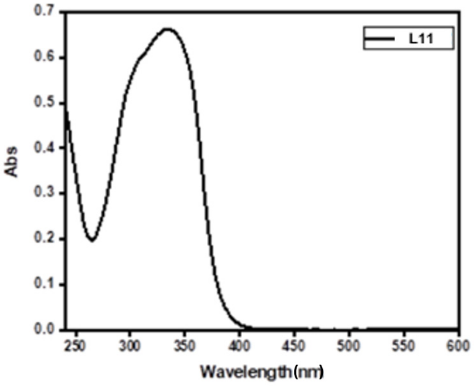

L12

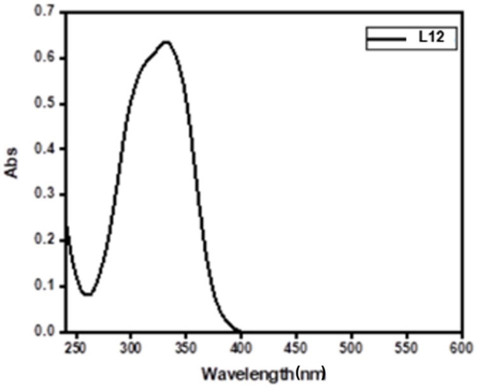

3. Fluorescence Spectra

Table S1. λ_{max} of emission at excitation 380 nm for compounds, compounds with Aβ fibrils, and compounds with soluble Aβ aggregates.

Compound	Compound λ_{em} (nm)	Compound+Fibrils λ_{em} (nm)	Compound+sAβ λ_{em} (nm)
**L1**	455	455	455
**L2**	460	440	440
**L3**	455	430	430
**L4**	455	435	435
**L5**	460	435	435
**L6**	455	430	430
**L7**	460	430	430
**L8**	460	430	430
**L9**	455	450	455
**L10**	455	435	435
**L11**	455	450	450
**L12**	450	435	430

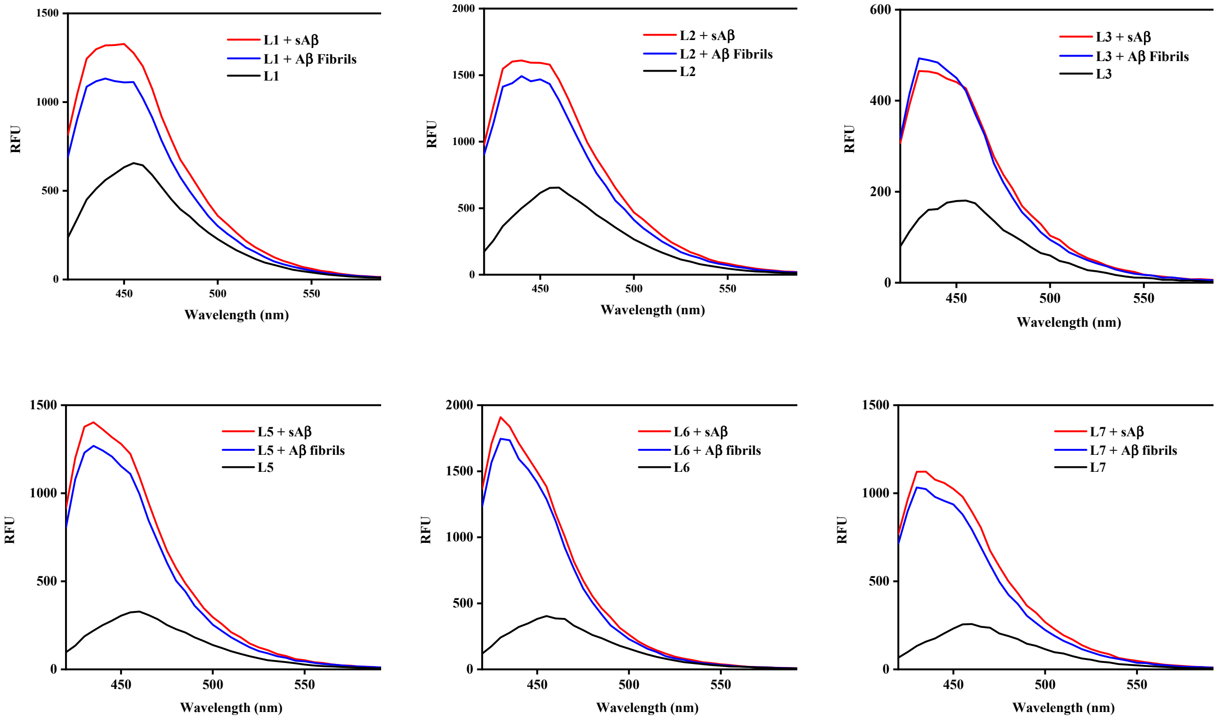

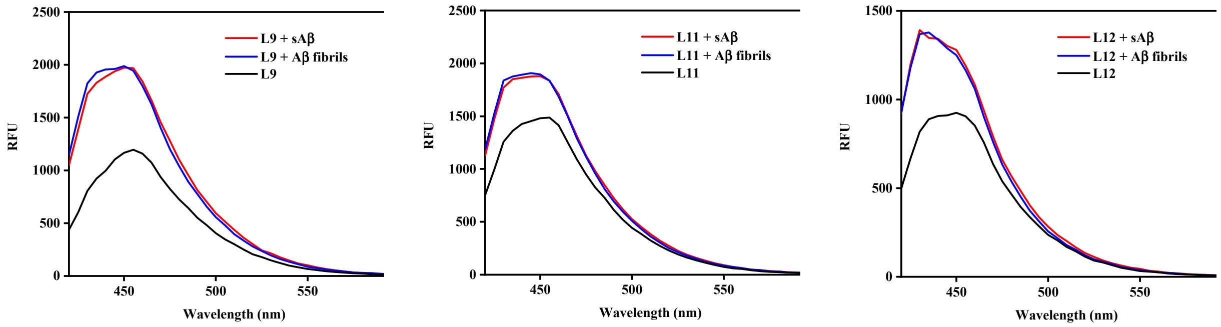

Figure S1. Fluorescence spectra of L1, L2, L3, L5, L6, L7, L9, L11, and L12 (black trace). Turn-on fluorescence of compounds with soluble (sAβ, red trace) and fibrillar (blue trace) Aβ aggregates.

4. Aβ_{40} Inhibition Assays

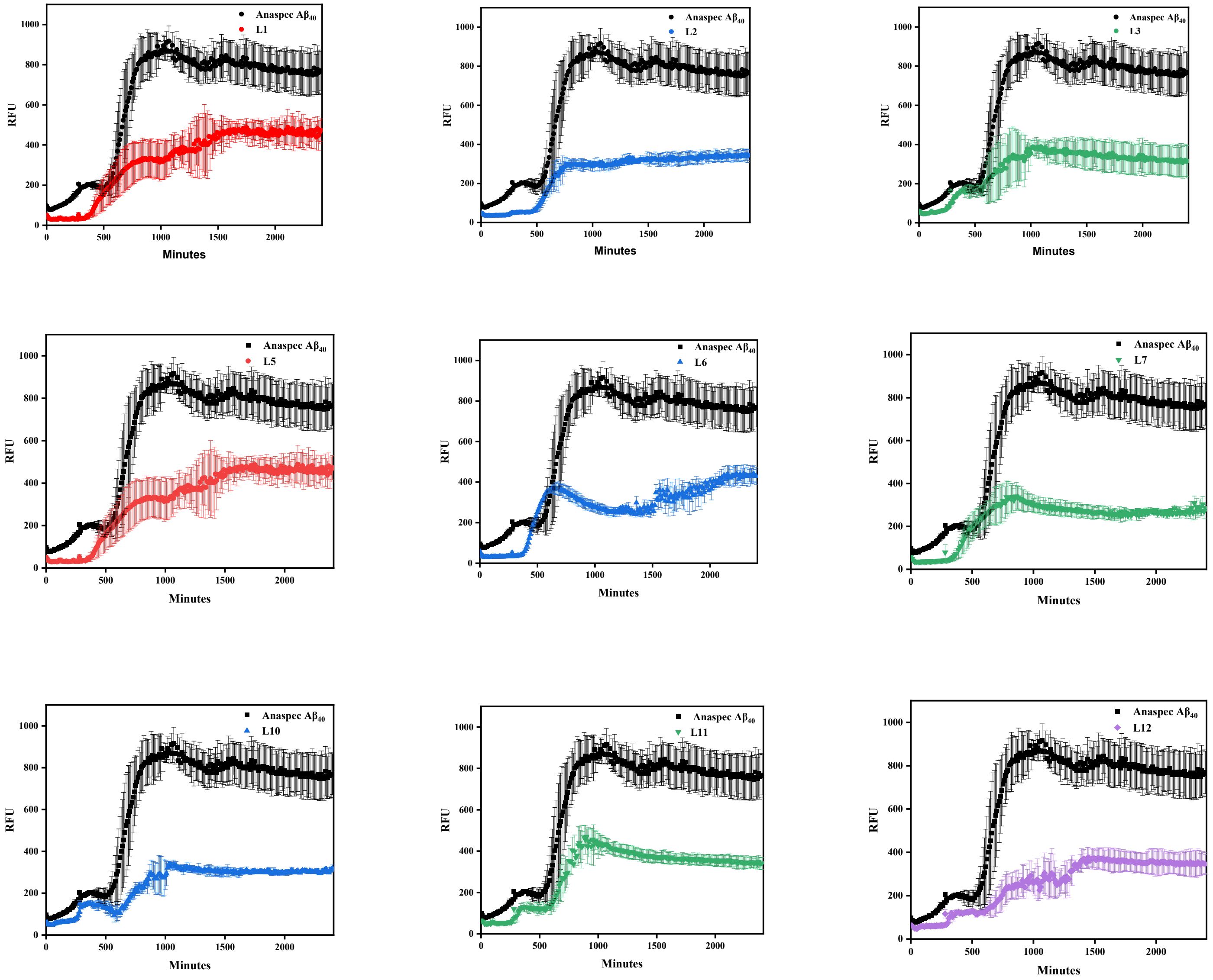

Figure S2. Aβ_{40} aggregation inhibition in presence of L1, L2, L3, L5, L6, L7, L10, L11, and L12 tracked in minutes from nucleation to fibrilization.

5. ThT Control Studies

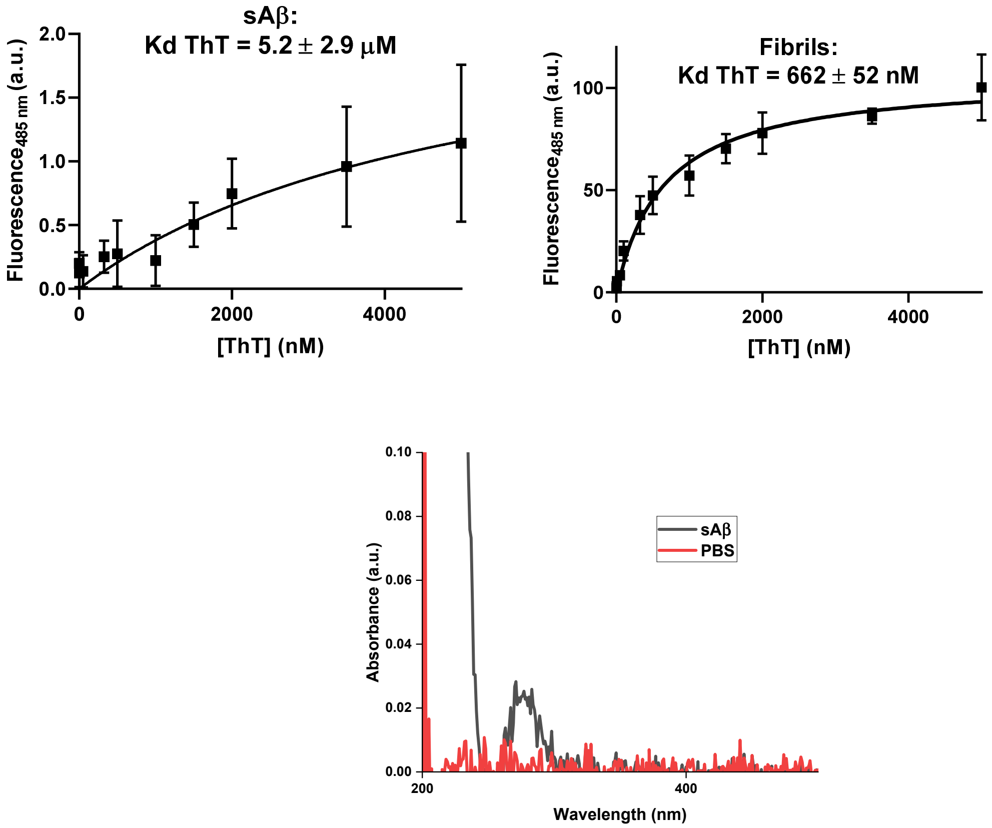

Figure S3. The $A\beta_{42}$ fluorescence turn-on assay with ThT (em = 485 nm, ex = 435 nm) for soluble $A\beta_{42}$ and $A\beta_{42}$ fibrils was used to control for soluble vs. insoluble amyloid. [$A\beta_{42}$] = 5 μM; [ThT] = 1, 5, 10, 50, 100, 325, 500, 1000, 1500, 2000, 3500, 5000 nM). Using the protocol from the main text to prepare soluble and fibrillar $A\beta_{42}$, the UV-vis absorbance was measured for the soluble $A\beta_{42}$ used in the control. In a cuvette, 100 μL of [$A\beta_{42}$] = 20 μM. Soluble $A\beta_{42}$ control shows $A\beta_{42}$ signature absorbance band at 280 nm (bottom) which confirms the existence of amyloid species for soluble amyloid.[1] The K_d was taken for both fibrils and soluble amyloid with ThT. ThT does not bind to non-amyloids and will not exhibit a fluorescence enhancement. Soluble $A\beta_{42}$ control shows negligible turn-on (top left) compared to the fibrillar control, 6 nM, (top right) which matches the reported K_d for ThT and $A\beta_{42}$.[2]

6. Molecular Docking Data

Table S2. Molecular docking summary for 5OQV docked compounds. Ranking based on docking score.

Compound	Docking Score	Glide e-model (kcal/mol)
**L7**	-7.749	-72.099
**L11**	-7.749	-53.999
**L5**	-7.708	-71.690
**L6**	-7.696	-69.227
**L8**	-7.515	-64.145
**L12**	-7.374	-54.522
**L1**	-7.239	-67.812
**L4**	-7.140	-62.917
**L10**	-6.975	-66.622
**L9**	-6.943	-65.236
**L2**	-6.685	-62.721
**L3**	-6.681	-61.783

Table S3. Molecular docking summary for 6RHY docked compounds. Ranking based on docking score.

Compound	Docking Score	Glide e-model (kcal/mol)
**L2**	-5.055	-46.608
**L6**	-4.572	-45.696
**L3**	-4.395	-52.13
**L7**	-4.312	-49.4
**L4**	-4.132	-49.385
**L10**	-4.123	-45.857
**L12**	-3.97	-48.282
**L9**	-3.967	-48.275
**L5**	-3.961	-45.516
**L11**	-3.776	-50.625
**L1**	-3.436	-42.099
**L8**	-3.126	-38.738

7. Log D Measurements

Table S4. Log D values for the compounds.

Compounds	Log D
**L1**	1.33 ± 0.01
**L2**	1.48 ± 0.02
**L3**	0.98 ± 0.01
**L4**	1.44 ± 0.01
**L5**	1.39 ± 0.01
**L6**	1.37 ± 0.01
**L7**	1.35 ± 0.02
**L8**	1.29 ± 0.01
**L9**	0.93 ± 0.02
**L10**	1.03 ± 0.01
**L11**	0.85 ± 0.01
**L12**	0.81 ± 0.01

8. Hammett Parameter Approximations

Table S5. Hammett parameter approximations using web tool ertlmolecular.[3] In the software, R was to denote the stilbene framework. The EAGs are shown to be of moderate strength. The summation of σ for all the substituents on the EAG is given as approximation. There is only one substituent on the EDG for these compounds. A more negative value indicates a more electron-donating character.

Compounds	EDG σ_{total}	EAG $\sigma_{subtotal}$	$\Sigma\sigma_{total}$
**L1**	σ_p = -0.685	σ_p = -0.325<br>$\sigma_{m(tacn)}$ = 0.005<br>$\sigma_{m(OMe)}$ = 0.029	σ_{tot} = -0.291
**L2**	σ_p = -0.624	σ_p = -0.325<br>$\sigma_{m(tacn)}$ = 0.005<br>$\sigma_{m(OMe)}$ = 0.029	σ_{tot} = -0.291
**L3**	σ_p = -0.624	σ_p = -0.325<br>$\sigma_{m(tacn)}$ = 0.005	σ_{tot} = -0.320
**L4**	σ_p = -0.685	σ_p = -0.325<br>$\sigma_{m(tacn)}$ = 0.005	σ_{tot} = -0.320
**L5**	σ_p = -0.627	σ_p = -0.325<br>$\sigma_{m(tacn)}$ = 0.005<br>$\sigma_{m(OMe)}$ = 0.029	σ_{tot} = -0.291
**L6**	σ_p = -0.627	σ_p = -0.325<br>$\sigma_{m(tacn)}$ = 0.005	σ_{tot} = -0.320
**L7**	σ_p = -0.594	σ_p = -0.325<br>$\sigma_{m(tacn)}$ = 0.005<br>$\sigma_{m(OMe)}$ = 0.029	σ_{tot} = -0.291
**L8**	σ_p = -0.594	σ_p = -0.325<br>$\sigma_{m(tacn)}$ = 0.005	σ_{tot} = -0.320
**L9**	σ_p = -0.476	σ_p = -0.325<br>$\sigma_{m(tacn)}$ = 0.005	σ_{tot} = -0.291

		$\sigma_{m(OMe)}$ = 0.029	
**L10**	σ_p = -0.476	σ_p = -0.325<br>$\sigma_{m(tacn)}$ = 0.005	σ_{tot} = -0.320
**L11**	σ_p = -0.631	σ_p = -0.325<br>$\sigma_{m(tacn)}$ = 0.005<br>$\sigma_{m(OMe)}$ = 0.029	σ_{tot} = -0.291
**L12**	σ_p = -0.631	σ_p = -0.325<br>$\sigma_{m(tacn)}$ = 0.005	σ_{tot} = -0.320

9. Molecular Docking Images

5OQV

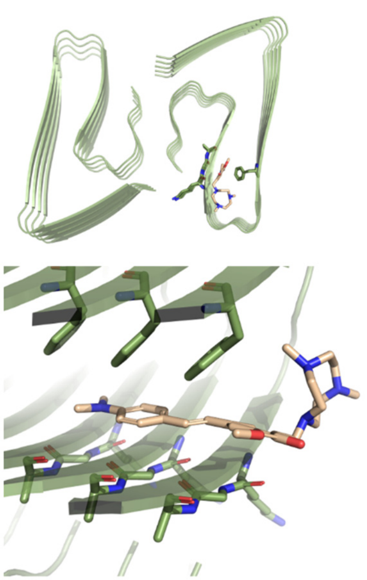

L1

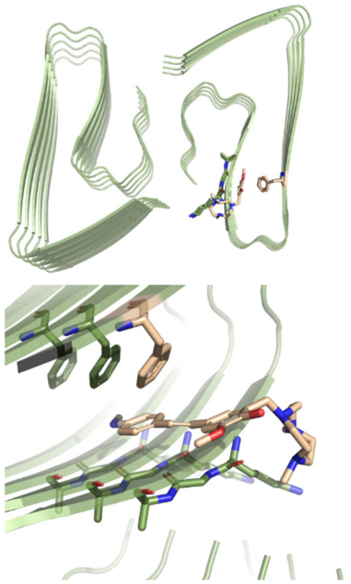

L2

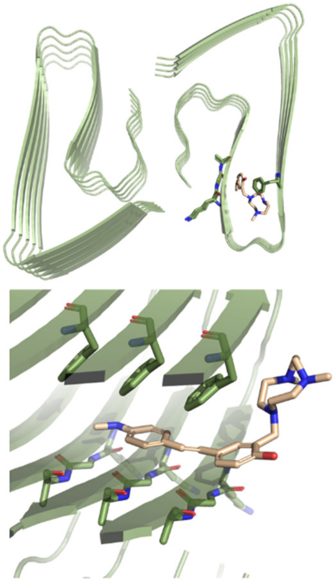

L3

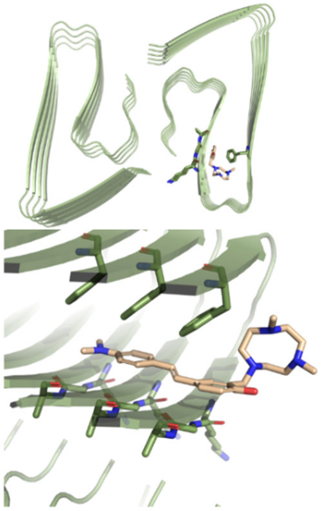

L4

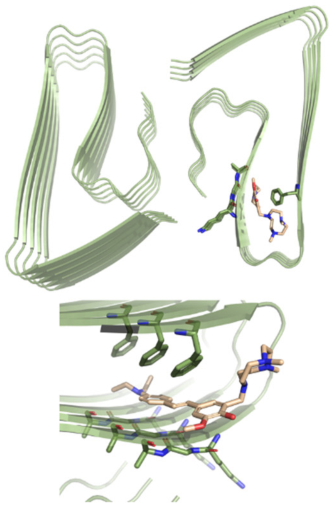

L5

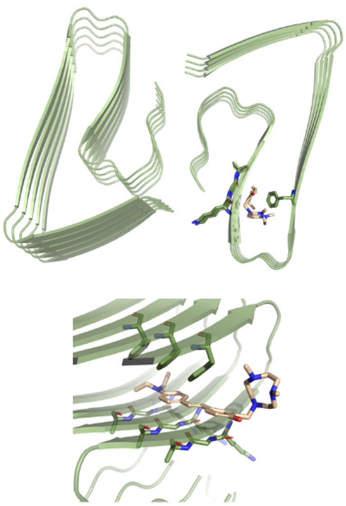

L6

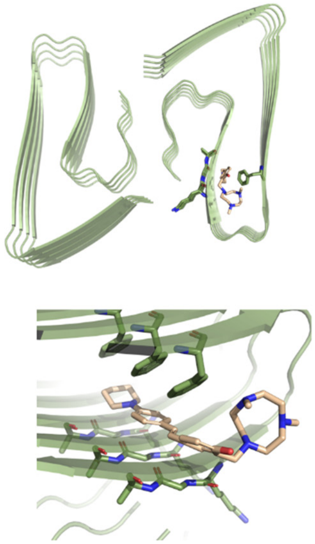

L8

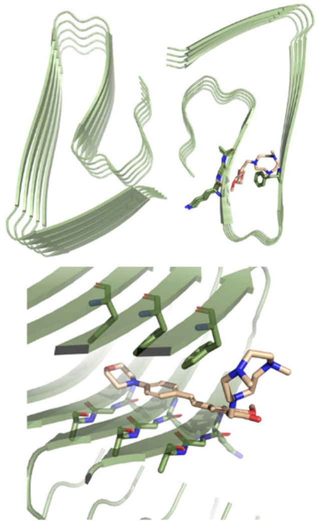

L9

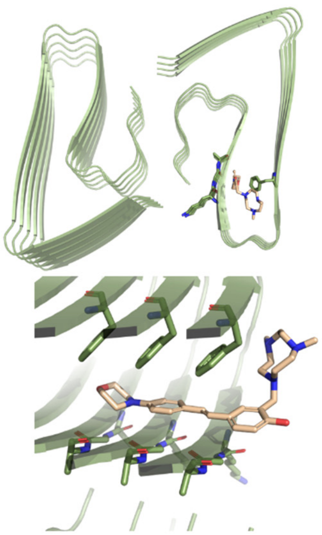

L10

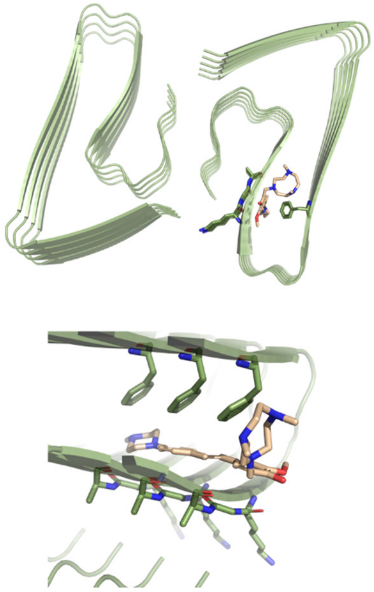

L11

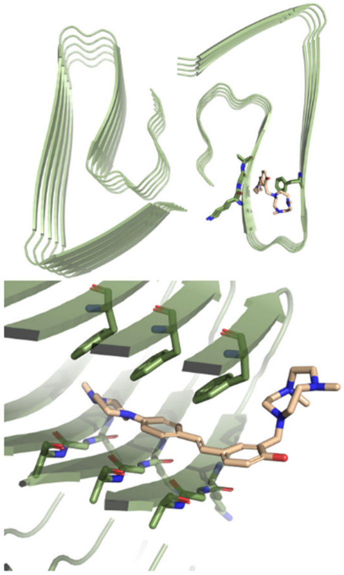

L12

6RHY

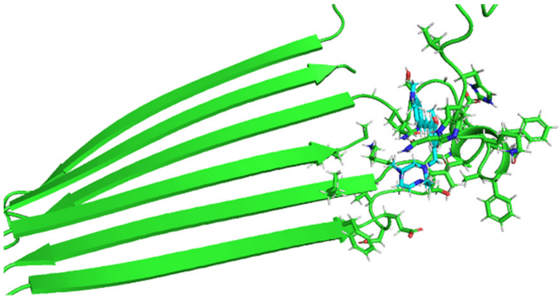

L1

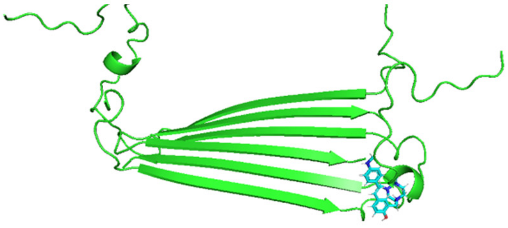

L3

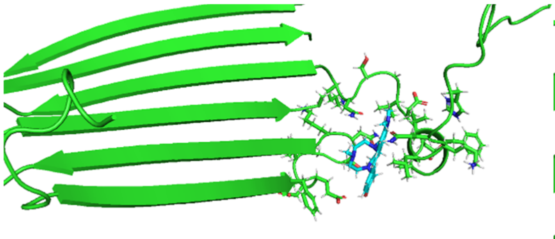

L4

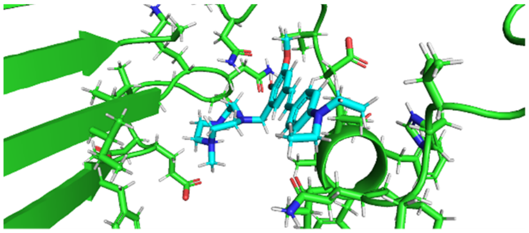

L5

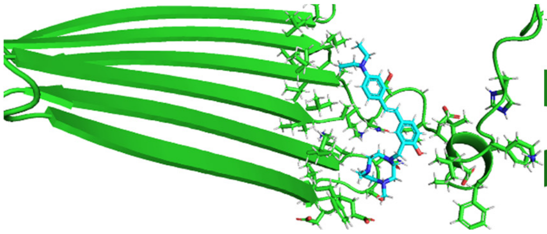

L6

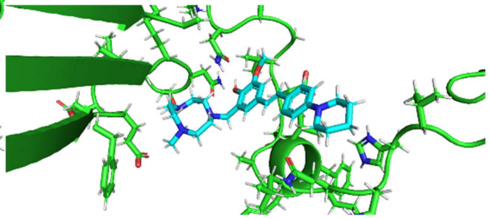

L7

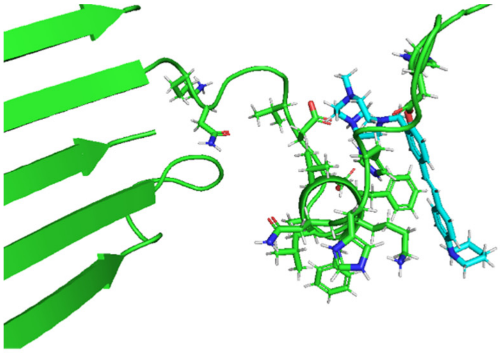

L8

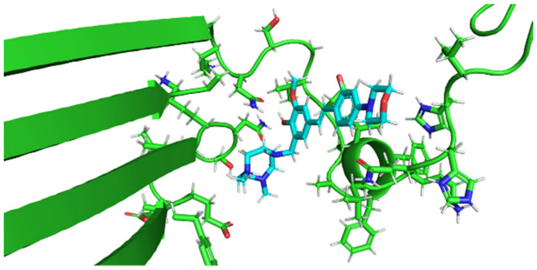

L9

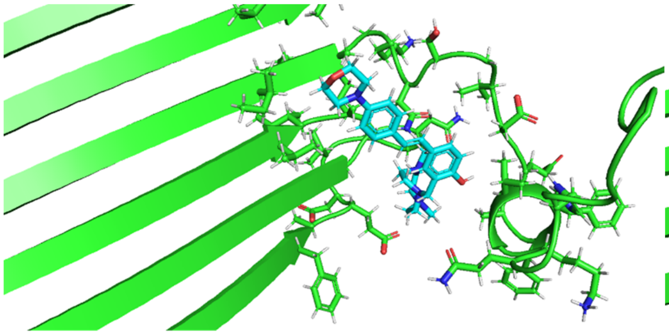

L10

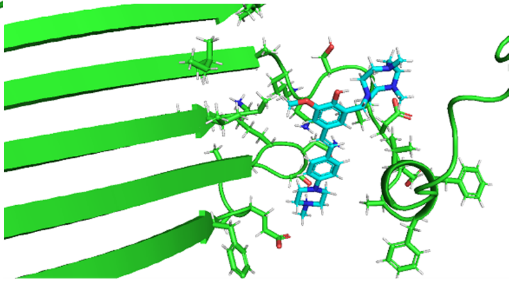

L11

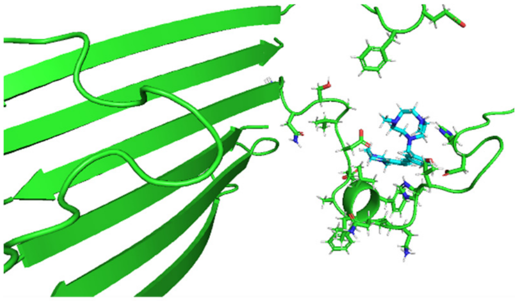

L12

10. References

1. Xue, C.; Lee, Y. K.; Tran, J.; Chang, D.; Guo, Z., A mix-and-click method to measure amyloid-β concentration with sub-micromolar sensitivity. *R. Soc. Open Sci.* **2017,** *4* (8), 170325.
2. Groenning, M., Binding mode of Thioflavin T and other molecular probes in the context of amyloid fibrils—current status. *J. Chem. Biol.* **2010,** *3* (1), 1-18.
3. Ertl, P., A Web Tool for Calculating Substituent Descriptors Compatible with Hammett Sigma Constants. *Chemistry–Methods* **2022,** *2* (12), e202200041.
